# Gut dysbiosis impairs HPA axis function and exacerbates post-stroke inflammation in high-fat diet-fed male mice

**DOI:** 10.1128/spectrum.03549-25

**Published:** 2026-08-21

**Authors:** Hanlin Zhong, Zeping Jin, Yiqi Liu, Yang Wang, Yunpeng Liu

**Affiliations:** 1 Department of Neurosurgery, Beijing Chao-Yang Hospital, Capital Medical University 12517, Beijing, Beijing, China; National Center for Genetic, Pathum Thani, Thailand; Bangabandhu Sheikh Mujibur Rahman Science and Technology University, Gopalganj, Bangladesh

**Keywords:** high-fat diet, gut microbiota, hypothalamic–pituitary–adrenal axis, ischemic stroke, *Akkermansia muciniphila*

## Abstract

High-fat diet (HFD)-induced metabolic stress is associated with impaired hypothalamic–pituitary–adrenal (HPA) axis function, but its impact on stroke outcomes and the role of the gut microbiota remain unclear. In a mouse middle cerebral artery occlusion (MCAO) model, we found that HFD induced gut dysbiosis, suppressed hypothalamic CRH expression, and reduced circulating corticosterone, which in turn exacerbated neuroinflammation and neuronal apoptosis, and worsened neurological recovery. Interventions with fecal microbiota transplantation or supplementation of *Akkermansia muciniphila* partially restored HPA axis activity, mitigated inflammatory injury, and improved behavioral outcomes post-stroke. These findings suggest that a gut microbiota–HPA axis may be relevant under metabolic stress conditions and open a possible microbiota-based adjunctive strategy for improving stroke prognosis in metabolically vulnerable populations.

**IMPORTANCE:**

Metabolic disorders such as obesity and high-fat diet exposure are known to worsen stroke outcomes, but the mechanisms connecting metabolic stress, gut microbiota alterations, and neuroendocrine dysfunction have not been well defined. Our data indicate that gut dysbiosis under HFD conditions is associated with HPA axis suppression and worse neuroinflammatory injury after stroke and that microbiota-targeted intervention can restore hormonal regulation and functional recovery. This highlights the gut microbiota–neuroendocrine interface as a promising area for therapeutic exploration in metabolically at-risk stroke patients.

## INTRODUCTION

Ischemic stroke, a leading cause of mortality and long-term disability worldwide (1), involves not only cerebrovascular pathology but also complex neuroendocrine responses, and increasing attention has been paid to the role of endocrine mechanisms in its pathophysiology (2). Among these, the hypothalamic–pituitary–adrenal (HPA) axis has been suggested to play a critical regulatory role. However, whether hormone therapy should be employed in stroke management remains controversial (3, 4). While multiple studies have indicated that high-dose glucocorticoid therapy does not offer significant clinical benefit and may even be associated with adverse outcomes such as hyperglycemia and immunosuppression (5), the inconsistent clinical efficacy of corticosteroid treatment in acute ischemic stroke means that a definitive causal link between elevated hormone levels and poor prognosis has not been established. Conversely, preclinical evidence suggests that glucocorticoids may exert neuroprotective effects by reducing infarct volume, preserving blood-brain barrier integrity, and modulating inflammation (6, 7). A recent large-scale clinical study further indicated that certain patient subgroups might benefit from hormone-based interventions (8, 9). Nonetheless, due to inconsistent findings and limited overall efficacy, no standardized therapeutic consensus has been reached. This inconsistency highlights the need to better define which patients might benefit from hormone therapy. We propose that individual baseline hormonal status may serve as a key determinant. In patients with impaired HPA axis function and sub-physiological glucocorticoid levels, moderate hormonal restoration may provide clinical benefit (10). In contrast, exogenous hormone supplementation in individuals with normal or elevated baseline levels may result in excessive glucocorticoid exposure, triggering negative feedback on endogenous secretion and disrupting systemic homeostasis (11).

Recent studies have established that high-fat diet (HFD), a major driver of metabolic and cardiovascular disorders, is closely linked to neuroendocrine dysregulation and adverse stroke outcomes. Beyond inducing metabolic inflammation and insulin resistance, chronic HFD consumption has been shown to alter the activity of the HPA axis, disrupt glucocorticoid signaling, and potentiate neuroinflammatory cascades that exacerbate ischemic injury (12, 13). Although some studies have reported increased basal or stress-induced glucocorticoid output after HFD exposure, others have observed blunted HPA axis responsiveness or reduced corticosterone levels under prolonged metabolic stress; therefore, inconsistencies remain regarding the direction and extent of these hormonal changes (14, 15). Moreover, obesity and metabolic syndrome—key consequences of chronic HFD—have been increasingly recognized as major risk factors for both gut microbiota dysbiosis and cerebrovascular pathology (16). Numerous studies have demonstrated that gut microbial imbalance contributes to endothelial dysfunction, systemic inflammation, and altered neurovascular coupling, thereby increasing susceptibility to stroke and hindering recovery (17, 18). The gut–brain axis provides a mechanistic framework linking metabolic signals, neuroendocrine stress responses, and neural injury. Within this framework, the HPA axis serves as a key neuroendocrine pathway through which peripheral metabolic and microbial cues influence central nervous system homeostasis (19–22). However, direct experimental evidence delineating how HFD-induced gut microbiota alterations impact the HPA axis and stroke outcomes remains limited.

To address these gaps, the present study was designed to examine whether microbiota-targeted interventions in HFD-fed mice are associated with changes in HPA axis activity, endogenous hormone levels, and post-stroke outcomes, thereby providing partial mechanistic insight into the gut microbiota–HPA axis–stroke relationship. Among gut microbes, *Akkermansia muciniphila*, a mucin-degrading bacterium enriched in healthy individuals, has emerged as an important regulator of metabolic homeostasis and intestinal barrier integrity (23). Previous studies have shown that *Akkermansia* supplementation can ameliorate obesity-related metabolic dysfunction, reduce inflammation, and modulate gut–brain signaling, including HPA axis responses under stress-related conditions (24–26). Therefore, we hypothesized that *Akkermansia* might serve as a candidate microbial intervention capable of restoring neuroendocrine balance and mitigating stroke-related injury under high-fat dietary conditions.

This work aims to deepen our mechanistic understanding of gut microbiota–neuroendocrine interactions and to offer a potentially precise and controllable adjunctive strategy warranting further preclinical and clinical evaluation.

## MATERIALS AND METHODS

### Experimental design and workflow

**Fig 1:**
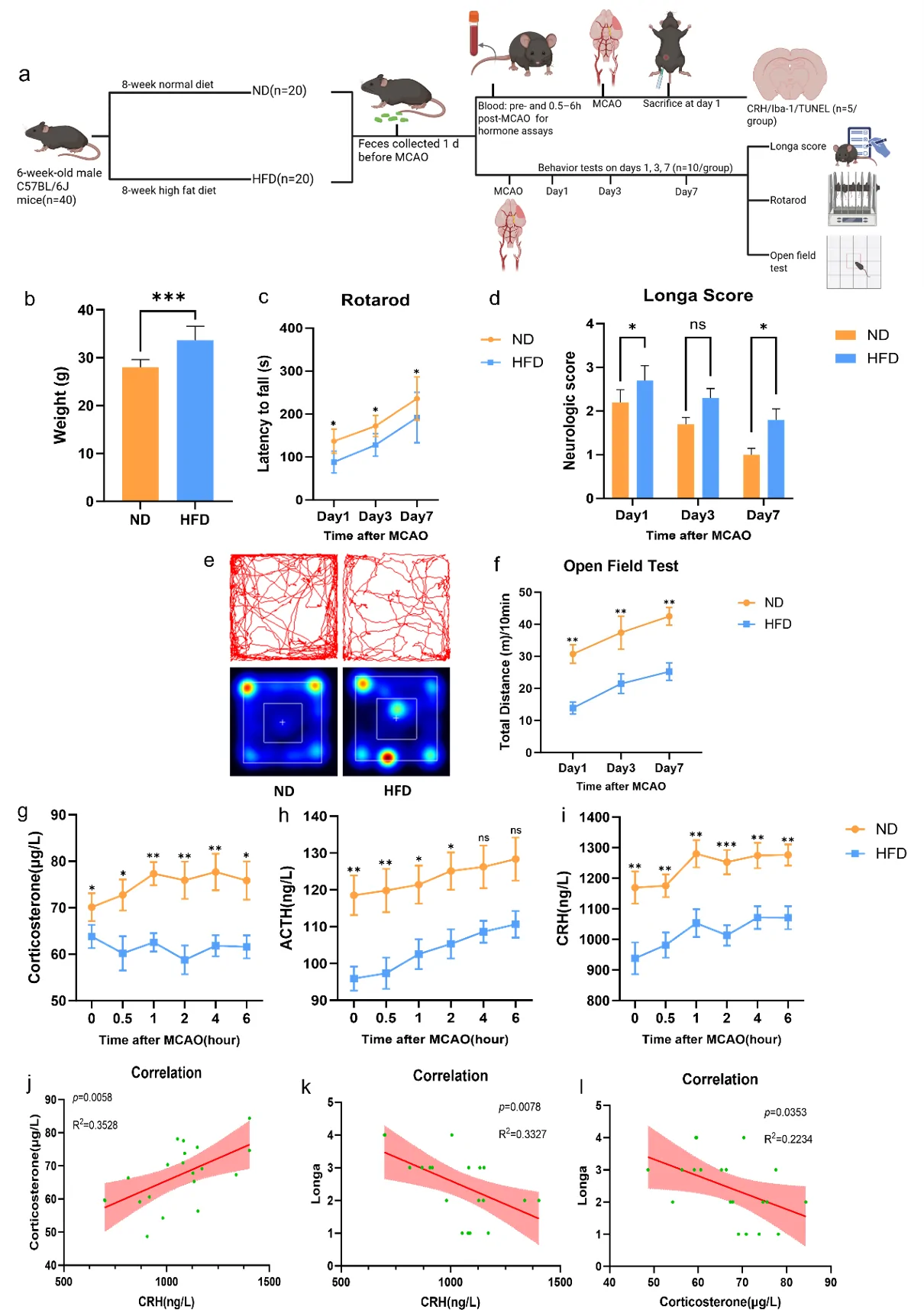
Experimental design and effects of high-fat diet on post-stroke outcomes and HPA axis function. (**a**) Schematic illustration of the overall experimental workflow, showing two independent cohorts for behavioral and histological analyses. (**b**) Increased body weight in HFD-fed mice. (**c**) Rotarod performance (latency to fall) at post-MCAO days 1, 3, and 7. (**d**) Neurological deficit scores (Longa score) post-MCAO. (**e**) Trajectory diagrams and heatmaps of the open field test on day 1 post-MCAO. (**f**) Total movement distance in the open field test over 10 min. (**g–i**) Serum corticosterone, ACTH, and CRH levels at baseline (0, pre-MCAO) and 0.5, 1, 2, 4, and 6 h post-occlusion. (**j**) Positive correlation between corticosterone and CRH levels (*P* = 0.0058, *R*² = 0.3528). (**k, l**) Negative correlations between CRH/corticosterone levels and Longa scores (*P* = 0.0078/0.0353, *R*² = 0.3327/0.2234). Data represent mean ± SEM. Two-way ANOVA with Tukey’s post hoc test (**c, d, g–i**); unpaired *t*-test (**b**); Spearman correlation **(j–l**). *n* = 10 per group. *, *P* < 0.05; **, *P* < 0.01; ***, *P* < 0.001; ns, not significant.

A schematic overview of the experimental workflow is shown in Fig. 1a and 4a.

The study consisted of two main parts. In the first part, mice fed a normal diet (ND) or a high-fat diet (HFD) for 8 weeks underwent MCAO to examine the effects of HFD on stroke outcomes and HPA axis activity. Because the primary aim was to examine the effects of HFD and microbiota intervention under ischemic conditions, no separate sham-operated group was included. Blood samples for hormone assays were collected before MCAO and at 0.5, 1, 2, 4, and 6 h after occlusion to evaluate dynamic hormonal responses to ischemic stress.

In the second part, HFD-fed mice received daily oral gavage for 7 consecutive days with fecal microbiota transplantation (FMT), *Akkermansia muciniphila* (AKK), or phosphate-buffered saline (PBS) before MCAO to test whether microbiota modulation could restore HPA axis function. Only pre-MCAO blood samples were collected in this part, as the primary aim was to assess hormone levels after microbiota intervention rather than post-ischemic fluctuations.

For each experimental group (ND, HFD, HFD^PBS^, HFD^FMT^, and HFD^AKK^), behavioral and histology/biochemistry analyses were performed in separate subgroups of mice, with *n* = 10 per group for each subgroup. The reported sample sizes represent the final total number of animals included in each analysis, rather than numbers per repeated cohort. The behavioral subgroup was used for neurological scoring, rotarod, and open-field tests at 1, 3, and 7 days after reperfusion, whereas the histology subgroup was used for blood collection and brain tissue analyses on day 1 after MCAO. Among the histology subgroup, five mice were used for immunofluorescence staining (CRH and Iba-1) and five for terminal deoxynucleotidyl transferase (TUNEL) staining. Fecal samples were collected 1 day before MCAO, and 12 mice per group were randomly selected for 16S rRNA sequencing to analyze gut microbiota composition. Some animals therefore contributed both microbiome data and behavioral or histological/biochemical data, but fecal collection was performed before MCAO and did not interfere with subsequent outcome assessments.

### Animals and ethics statement

Six-week-old C57BL/6J mice (18–22 g at baseline; Vital River Laboratory Animal Technology, Beijing, China) were used. Only male mice were used in this study to reduce potential variability associated with estrous cycle-related hormonal fluctuations; the implications of this sex limitation are discussed in the “Limitations” section. Mice were housed six per individually ventilated cage, with controlled temperature, humidity, a 12-h light/dark cycle, and free access to food and water. Environmental enrichment (nesting material and shelters) was provided. Body weight was measured before MCAO surgery to confirm weight gain after the 8-week feeding period. All animal experiments were approved by the Animal Ethics Committee of Capital Medical University (Approval No. AEEI-2022-226). Animals were euthanized by an overdose of sodium pentobarbital (200 mg/kg, i.p.) at the designated endpoint. Animals were randomly allocated to experimental groups using a random number generator. Investigators conducting behavioral scoring, histological assessments, and statistical analyses were blinded to group allocation.

### Middle cerebral artery occlusion (MCAO)

To establish the MCAO model using the intraluminal filament method, mice were first anesthetized with isoflurane inhalation. A midline neck incision was then made to expose the left common carotid artery (CCA), external carotid artery (ECA), and internal carotid artery (ICA), followed by blunt dissection of surrounding connective tissues. After temporarily occluding the ICA and CCA, a monofilament (Model: MSMC23B008PK50, RWD Life Science Co.) was inserted into the ECA and advanced into the ICA until mild resistance was encountered. Following filament fixation, focal cerebral blood flow was analyzed using laser Doppler flowmetry. A reduction in cerebral blood flow to below 60% of the baseline level, maintained for 60 min, was considered indicative of successful model establishment. Across all MCAO experiments, 10 mice were excluded because of unsuccessful model establishment, and 11 mice were excluded because of death during the surgical procedure.

### Serum hormone enzyme-linked immunosorbent assay (ELISA)

For the determination of serum hormones and inflammatory factors, blood samples collected from mouse tail veins were gathered in clean centrifuge tubes. MCAO procedures were performed between 11:00 and 15:00, and blood samples were collected at predefined time points using the same schedule across groups. The specimens were immediately centrifuged at 3,000 rpm for 10 min, and the separated serum samples were stored at −80°C until analysis. Serum CRH, adrenocorticotropic hormone (ACTH), and corticosterone concentrations were quantified using an ELISA kit (Hengyuan Biological Technology Co., Shanghai, China) following the manufacturer’s protocols.

### Behavior testing procedures

#### Neurological score

Neurological deficits were evaluated 24 h post-reperfusion using the Longa score method: 0 (no deficit), 1 (forelimb flexion during tail suspension), 2 (consistent reduced resistance to lateral push), 3 (unidirectional circling toward the paretic side), 4 (critical impairment involving locomotor disability), and 5 (mortality).

#### Rotarod test

The rotarod test was conducted by placing mice on a rotating rod with constant acceleration from 0 to 300 rpm over 300 s. The latency to fall was recorded for each mouse, and three consecutive trials were performed with 15-min inter-trial intervals.

#### Open field test

The locomotor activity in mice was assessed using the open field test. Each mouse was placed in an open-field arena (50 cm × 50 cm × 30 cm) starting from the same initial position, and their movements were recorded for 15 min using a video tracking system. Data from the initial 5 min were excluded from analysis. The total distance traveled was quantified using EthoVision XT software (Noldus Information Technology, Wageningen, Netherlands).

### Gut microbiome analysis

Fecal sample collection was performed by elevating the tail and applying gentle abdominal pressure to stimulate defecation. Freshly expelled feces were collected using sterile forceps and immediately transferred into sterile cryovials, which were subsequently stored at −80°C. Each biological sample contained two to three fecal pellets. Gut microbiota profiling was conducted through 16S rRNA amplicon sequencing on the Illumina NovaSeq platform by Hebei LabMed Technology Co., Ltd. (Hebei, China). The V4 region of the bacterial 16S rRNA gene was amplified using barcoded 515F/806R primers. Paired-end reads were demultiplexed according to barcode and primer sequences, merged using FLASH v1.2.11, processed with Cutadapt to remove residual primer sequences, and quality-filtered using fastp v0.23.1. Chimeric sequences were removed by comparison with the reference database, and effective tags were denoised using the DADA2 module in QIIME2 to generate amplicon sequence variants. Taxonomic annotation was performed in QIIME2 against the SILVA 138.1 database. All samples were normalized to the minimum sequencing depth before alpha- and beta-diversity analyses.

### Immunofluorescence staining

Paraffin-embedded brain sections were sequentially dewaxed in three changes of eco-friendly dewaxing solution (G1128, Servicebio) and rehydrated through graded ethanol to distilled water. Antigen retrieval was performed by microwave heating in citrate buffer (pH 6.0, G1202, Servicebio) for 30 min, followed by cooling and three 5-min PBS washes (pH 7.4, G0002, Servicebio) on a decolorizing shaker. Tissue sections were encircled with a hydrophobic barrier pen (G6100, Servicebio), blocked with 3% bovine serum albumin (BSA) (GC305010, Servicebio) for 30 min, and incubated overnight at 4°C with primary antibodies: rabbit anti-CRH (1:500, 26-848-1-AP) and mouse anti-IBA1 (1:500, GB15105, Servicebio). After PBS washes, sections were incubated with CY3-conjugated goat anti-rabbit IgG (1:300, GB21303, Servicebio) and Alexa Fluor 488-conjugated goat anti-mouse IgG (1:400, GB25301, Servicebio) for 50 min at room temperature, protected from light. Nuclei were counterstained with DAPI (G1012, Servicebio) for 10 min, and tissue autofluorescence was quenched using reagent B (G1221, Servicebio). Slides were mounted with anti-fade medium (G1401, Servicebio) and imaged on a Nikon Eclipse C1 microscope with defined excitation/emission parameters (DAPI: 330–380/420 nm; Alexa Fluor 488: 465–495/515–555 nm; CY3: 510–560/590 nm). Fluorescence images were acquired using a 40× objective. For each marker, exposure time, gain, light intensity, and image resolution were kept constant across all groups within the same staining batch. For immunofluorescence quantification, three non-overlapping fields per animal were analyzed within predefined regions of interest. Fluorescence intensity was quantified using ImageJ by an investigator blinded to group allocation.

### TUNEL staining

Paraffin-embedded tissue sections were deparaffinized through sequential immersion in eco-friendly dewaxing solution (G1128, Servicebio) and rehydrated via graded ethanol series. Antigen retrieval was performed by incubating sections with proteinase K working solution (1:9 dilution in PBS, pH 7.4, Servicebio) at 37°C for 20 min, followed by three 5-min PBS (G0002, Servicebio) washes. After equilibration with reaction buffer, the TUNEL reaction mixture (TDT enzyme:dUTP:buffer = 2:5:50, Servicebio G1502) was applied to sections and incubated at 37°C for 1 h in a humidified chamber. Nuclei were counterstained with DAPI (G1012, Servicebio) for 10 min, and slides were mounted with anti-fade medium (G1401, Servicebio). Imaging was conducted using a Nikon Eclipse C1 fluorescence microscope. For TUNEL quantification, three non-overlapping fields per animal were analyzed in the infarct core. TUNEL-positive cells were expressed as a percentage of DAPI-positive nuclei, and analysis was performed blinded to group allocation.

### Fecal microbiota transplantation (FMT)

Fecal microbiota transplantation was performed to assess the effects of gut microbiota from ND-fed mice on HPA axis activity and stroke outcomes in HFD-fed mice. Fresh fecal pellets were collected daily from age-matched ND-fed donor mice (*n* = 5) and immediately resuspended in sterile PBS at a concentration of 100 mg/mL. The suspension was homogenized, filtered through a 70 μm nylon mesh to remove large particles, and administered to recipient HFD-fed mice by oral gavage at a volume of 200 μL per mouse. FMT was performed once daily for 7 consecutive days, starting 8 days prior to MCAO surgery. Recipient mice did not receive antibiotic pretreatment before FMT. The control group received equal volumes of sterile PBS without fecal material. Fecal samples were collected from recipient mice on the day before MCAO to assess microbial composition, and MCAO was induced 1 day after the final gavage.

### *Akkermansia muciniphila* (AKK) administration

*Akkermansia muciniphila* (BNCC341917) was obtained from the BeNa Culture Collection (Henan, China). Freshly cultured bacteria were harvested by centrifugation at 4,000 × *g* for 10 min and resuspended in sterile PBS to achieve a final concentration of 2 × 10⁹ colony-forming units (CFU) per 200 μL. HFD-fed mice received 200 μL of the AKK suspension (2 × 10⁹ CFU) by oral gavage once daily for 7 consecutive days immediately prior to MCAO surgery. Control mice were gavaged with 200 μL sterile PBS on the same schedule. The selected dosage (2 × 10⁹ CFU per 200 μL) was based on a previous study (27), in which this dose effectively modulated host metabolism and immune responses. The dose–response relationship was not examined in the present study.

### Statistical analysis

All data are presented as mean ± standard error of the mean (SEM). Statistical analyses were performed using GraphPad Prism 9.0 software (GraphPad Software, San Diego, CA, USA). Prior to hypothesis testing, data were assessed for normality using the Shapiro–Wilk test. For comparisons between two groups, unpaired two-tailed Student’s *t*-tests were used for normally distributed data, and Mann–Whitney U tests were applied for non-normally distributed data. For comparisons among more than two groups, one-way or two-way analysis of variance (ANOVA), followed by Tukey’s multiple comparison post hoc test, was employed. Behavioral outcomes (e.g., Longa scores, rotarod performance, and open field total distance) across time points were analyzed using two-way repeated measures ANOVA. Correlations between hormone levels and neurological scores were assessed using Spearman’s rank correlation coefficient. A *P* value < 0.05 was considered statistically significant.

## RESULTS

### High-fat diet worsens post-ischemic neurological outcomes and is associated with HPA axis dysfunction

To determine whether dietary regimens influenced neurological outcomes after MCAO, we first assessed functional deficits in mice fed different diets. As shown in Fig. 1a, HFD-fed mice exhibited a significant increase in body weight compared to ND controls (ND: 28.01 ± 0.47 g; HFD: 33.67 ± 0.85 g) after 8 weeks of dietary intervention. Neurological function was systematically assessed at days 1, 3, and 7 post-MCAO. The rotarod test revealed significantly impaired motor coordination and endurance in HFD mice compared to ND mice (Fig. 1b). Furthermore, Longa scores were higher in HFD mice than in ND mice at each post-stroke time point (Day 1: ND, 2.20 ± 0.29; HFD, 2.70 ± 0.33; Day 3: ND, 1.70 ± 0.15; HFD, 2.30 ± 0.21; Day 7: ND, 1.00 ± 0.15; HFD, 1.80 ± 0.25), indicating more severe neurological impairment in the HFD group (Fig. 1c). The open field test showed reduced locomotor activity in HFD mice, as evidenced by shorter total movement distances (Fig. 1d and e). These results indicate that HFD exacerbates post-ischemic neurological impairment compared to ND.

To investigate the impact of HFD on the HPA axis, blood samples were collected from the tail vein of mice before MCAO and at 0.5, 1, 2, 4, and 6 h post-occlusion for serum corticosterone, ACTH, and CRH quantification. HFD mice exhibited significantly lower levels of corticosterone, ACTH, and CRH levels at all time points compared to normal diet (ND) controls (Fig. 1f through h), indicating impaired HPA axis activity.

Correlation analyses revealed a significant positive relationship between serum corticosterone and CRH levels (*P* = 0.0058, *R*^2^ = 0.3528; Fig. 1i). Negative correlations were observed between CRH and Longa scores (*P* = 0.0078, *R*^2^ = 0.3327; Fig. 1j) and between corticosterone and Longa scores (*P* = 0.0353, *R*^2^ = 0.2234; Fig. 1k). Although these correlations were statistically significant, the *R*² values indicate that CRH and corticosterone levels explained only a modest proportion of the variance in neurological deficit scores.

### High-fat diet is associated with reduced CRH expression and exacerbated post-ischemic neuroinflammation and neuronal apoptosis

**Fig 2:**
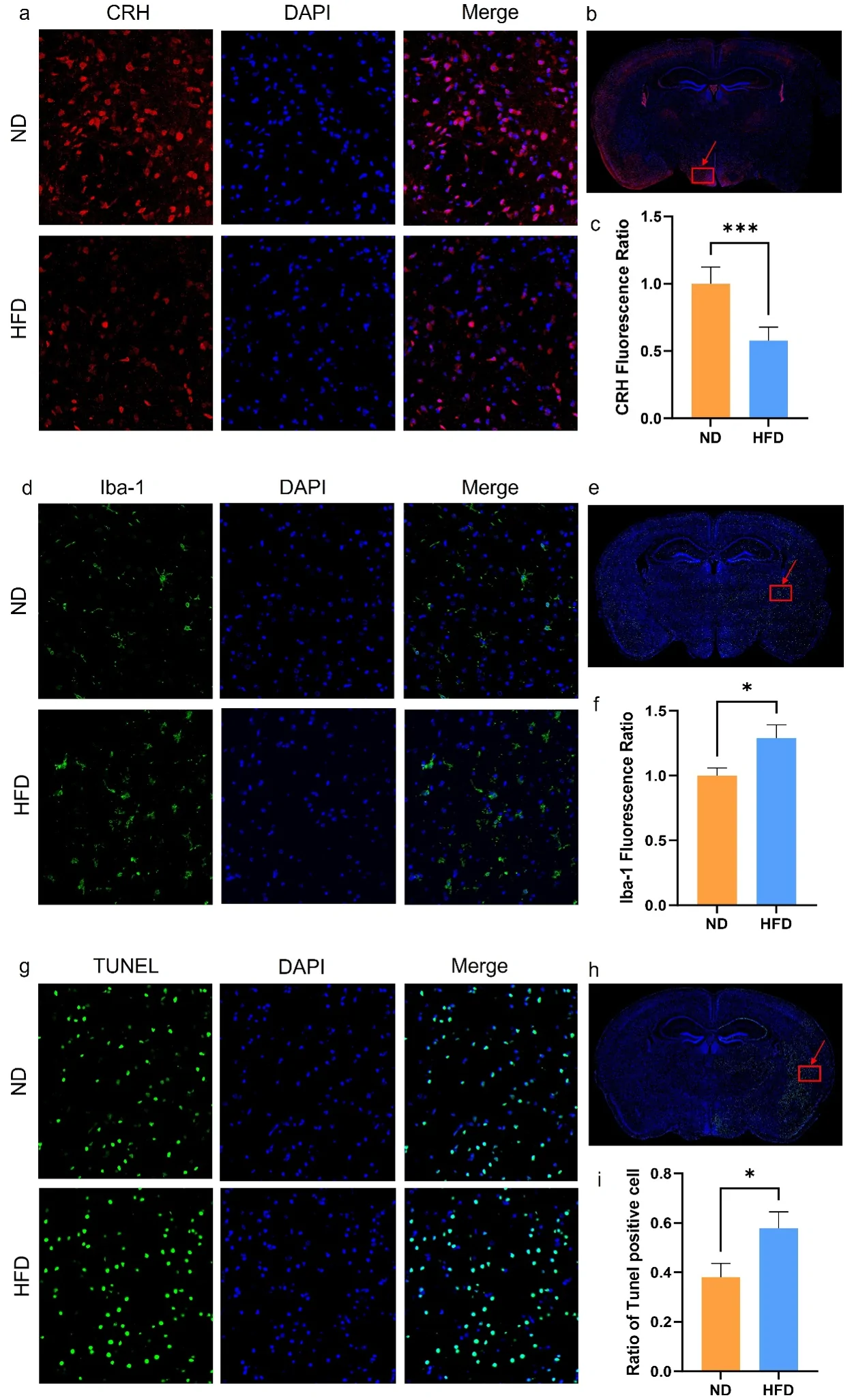
Immunofluorescence staining of CRH, Iba-1, and TUNEL following ischemic stroke. (**a**) Representative images of CRH IF staining (red) in the HFD and ND groups. (**b**) Analysis region: hypothalamus. (**c**) Quantification of mean fluorescence intensity of CRH in each group. (**d**) Representative images of Iba-1 IF staining (green) in the HFD and ND groups. (**e**) Analysis region: peri-infarct area. (**f**) Quantification of mean fluorescence intensity of Iba-1 in each group. (**g**) Representative images of TUNEL staining (green) in the HFD and ND groups. (**h**) Analysis region: infarct core. (**i**) Quantification of the proportion of TUNEL-positive cells in each group. Statistical analysis was performed using unpaired Student’s *t*-test; *n* = 5 per group. *, *P* < 0.05; **, *P* < 0.01; ***, *P* < 0.001.

Immunofluorescence staining for CRH in the hypothalamus revealed markedly reduced CRH expression in HFD-fed mice compared to those fed a normal diet. This observation suggests that HFD may impair the functional output of the HPA axis by downregulating hypothalamic CRH expression, potentially affecting stress responsiveness and hormonal regulation, but this analysis does not distinguish reduced expression per neuron from changes in CRH-positive cell number (Fig. 2a through c).

Given that immune cell activation is a critical pathological process following ischemic stroke and contributes to secondary neuronal damage, we next examined post-stroke neuroinflammatory responses under different dietary conditions. Microglial activation was assessed by measuring Iba-1 fluorescence intensity in the peri-infarct cortex. The HFD group exhibited significantly higher Iba-1 fluorescence intensity compared to the ND group (1.45 ± 0.12 vs 1.00 ± 0.06), indicating enhanced microglial activation and a more pronounced neuroinflammatory response in the post-ischemic brain following high-fat diet exposure (Fig. 2d through f).

To further evaluate the extent of neuronal injury, TUNEL staining was performed to quantify apoptotic cells in the infarct region. The number of TUNEL-positive cells was markedly elevated in HFD-fed mice relative to ND controls (0.67 ± 0.07 vs 0.47 ± 0.03), suggesting that HFD enhances neuronal apoptosis following ischemic insult. Collectively, these results demonstrate that HFD reduces the number of CRH-expressing hypothalamic neurons, amplifies neuroinflammatory responses, and promotes neuronal apoptosis in the context of ischemic stroke (Fig. 2g through i).

### High-fat diet alters gut microbial composition and reduces microbial diversity in mice

**Fig 3:**
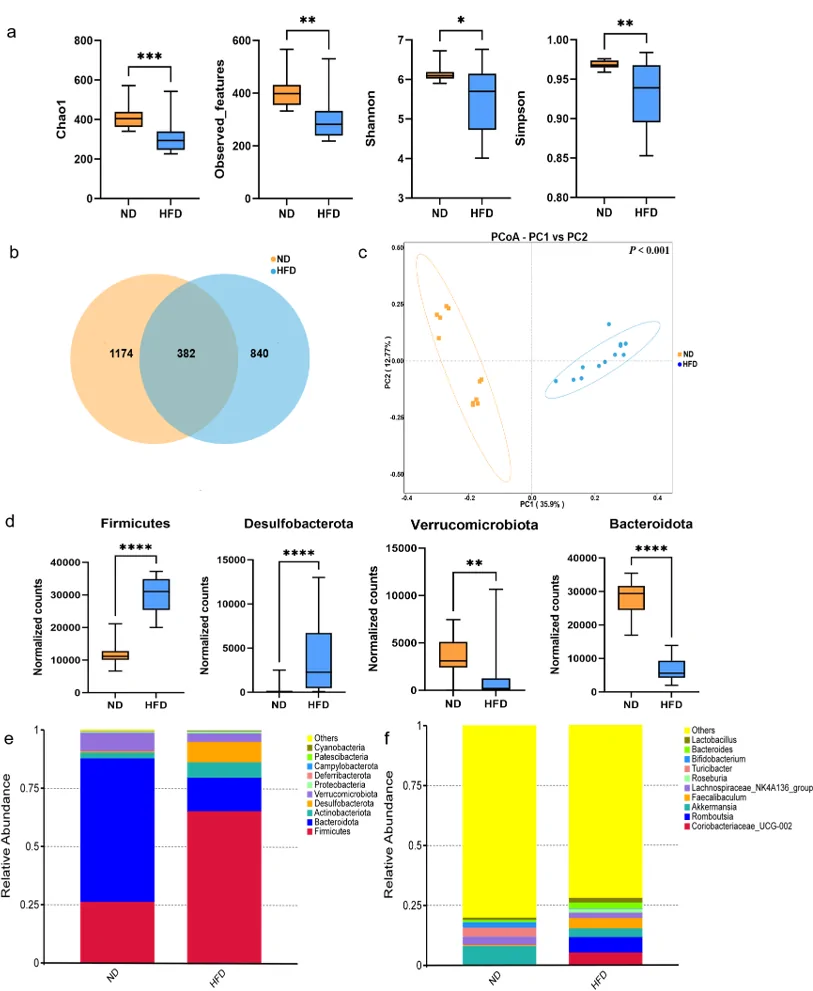
High-fat diet-induced alterations in gut microbiota diversity and taxonomic composition. (**a**) Alpha-diversity indices, including Chao1, observed features, Shannon, and Simpson, in ND and HFD mice. (**b**) Venn diagram showing shared and unique amplicon sequence variants (ASVs) between the ND and HFD groups. (**c**) Principal coordinate analysis (PCoA) showing separation of gut microbial communities between groups. (**d**) Normalized counts of representative bacterial phyla, including *Firmicutes*, *Desulfobacterota*, *Verrucomicrobiota*, and *Bacteroidota*. (**e, f**) Stacked bar plots showing relative microbial composition at the phylum and genus levels. *n* = 12 per group. *P < 0.05; **P < 0.01; ***P < 0.001; ****P < 0.0001.

To investigate the impact of a high-fat diet on the gut microbiota of mice, fecal samples were collected 1 day prior to MCAO surgery for 16S rRNA amplicon sequencing analysis. The Venn diagram revealed a total of 382 ASVs shared between the HFD and ND groups, with 1,174 unique ASVs identified in the ND group and 840 unique ASVs in the HFD group (Fig. 3b). Alpha diversity analysis indicated that microbial richness was notably reduced in HFD-fed mice compared to those on a normal diet, as reflected by lower Chao1 (*P* = 0.0004), observed features (*P* = 0.004), Shannon index (*P* = 0.016), and Simpson index (*P* = 0.008) (Fig. 3a). Beta diversity analysis further demonstrated significant differences in microbial community composition between the two groups (Fig. 3d). These results suggest that 8 weeks of HFD feeding markedly reduced both the richness and diversity of the gut microbiota in mice. Multilevel taxonomic analysis of microbial composition revealed that, at the phylum level, the relative abundances of *Firmicutes*, *Actinobacteria*, and *Desulfobacterota* were significantly increased in the HFD group, whereas the abundances of *Verrucomicrobiota and Lactobacillota* were significantly decreased compared to the ND group (Fig. 3d and e). Further analysis at the genus level showed a significant reduction in the abundance of *Akkermansia* in the HFD group relative to the ND group (Fig. 3d and f). Collectively, these findings indicate that the gut microbial composition in HFD-fed mice differs markedly from that in mice maintained on a normal diet.

### Gut microbiota intervention enhances HPA axis hormone levels and improves post-stroke functional recovery in HFD-fed mice

**Fig 4:**
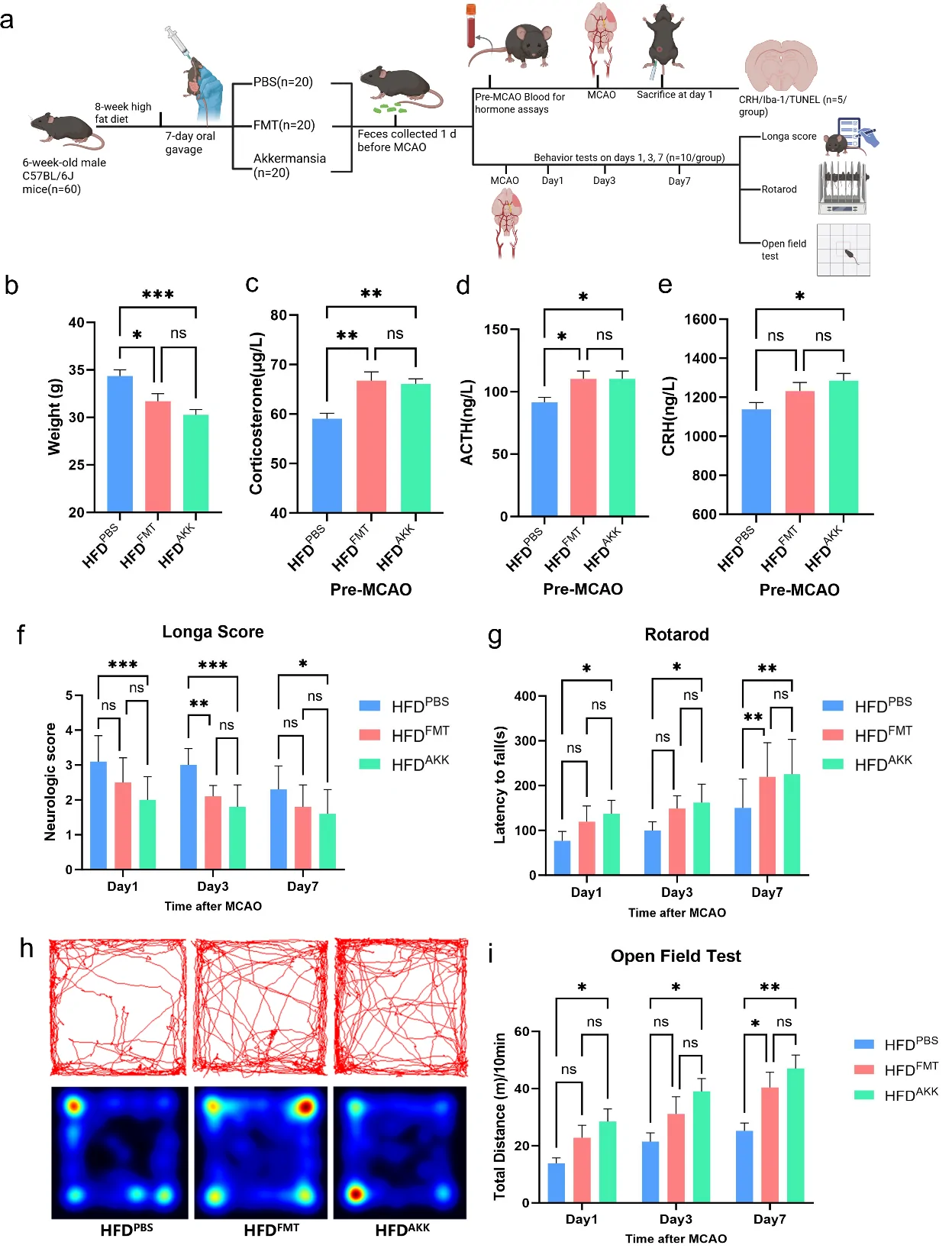
Gut microbiota intervention restores HPA axis function and improves post-stroke recovery in HFD-fed mice. (**a**) Schematic illustration of the experimental design showing 8-week HFD feeding, 7-day oral gavage with PBS, FMT, or AKK, followed by MCAO surgery. Fecal samples were collected 1 day before MCAO for microbiota analysis. Blood was collected before MCAO for hormone assays, and brain tissues were harvested on day 1 post-MCAO for CRH, Iba-1, and TUNEL staining. Behavioral tests (Longa score, rotarod, and open field) were performed at 1, 3, and 7 days after reperfusion. (**b**) Body weight before MCAO. (**c–e**) Serum corticosterone, ACTH, and CRH levels measured before MCAO. (**f**) Neurological deficit scores (Longa score) at days 1, 3, and 7 post-MCAO. (**g**) Rotarod performance (latency to fall) at the same time points. (**h**) Representative trajectory plots and heatmaps of the open field test on day 1 post-MCAO. (**i**) Total movement distance in the open field test over 10 min. Data are presented as mean ± SEM. Two-way ANOVA with Tukey’s post hoc test (**f–i**); one-way ANOVA with Tukey’s post hoc test (**b–e**). *n* = 10 per group. *, *P* < 0.05; **, *P* < 0.01; ***, *P* < 0.001; ns, not significant.

To examine whether gut microbiota–targeted interventions could influence hypothalamic–pituitary–adrenal (HPA) axis activity and neurological outcomes following ischemic stroke, two approaches were used. In the first, fecal microbiota from ND-fed mice were transplanted into HFD-fed recipients for 7 consecutive days (HFD^FMT^ group). In the second, the differential strain *Akkermansia muciniphila*, identified by 16S rRNA sequencing, was orally administered to HFD-fed mice at a dose of 2 × 10⁹ CFU/day for 7 days (HFD^AKK^ group). The control group (HFD^PBS^) received an equal volume of sterile PBS. All interventions began 8 days prior to MCAO, and the final gavage was administered 1 day before surgery (Fig. 4a).

Body weight measured immediately before MCAO was significantly reduced in both the HFD^FMT^ and HFD^AKK^ groups compared with the HFD^PBS^ group (Fig. 4b). Because both FMT and AKK administration reduced body weight in HFD mice, the subsequent improvements in hormone levels and stroke outcomes should be interpreted in the context of concomitant metabolic changes, rather than being attributed solely to microbiota–HPA axis modulation.

To determine whether microbiota intervention affected HPA axis activity, serum levels of CRH, ACTH, and corticosterone were measured from tail-vein blood collected 1 day before MCAO. As shown in Fig. 4c through e, both FMT and AKK treatments increased circulating hormone levels compared with PBS controls. Specifically, ACTH and corticosterone concentrations were significantly elevated in both intervention groups, whereas CRH showed an upward trend without reaching statistical significance in the HFD^FMT^ group. Thus, FMT appeared to partially restore the HPA axis hormone profile, with more robust effects on ACTH and corticosterone than on serum CRH. No significant differences were detected between the HFD^FMT^ and HFD^AKK^ groups for any of the three hormones.

Although elevated HPA axis hormones accompanied functional improvement, the present data do not establish a causal relationship between hormone restoration and behavioral recovery. Therefore, the improved outcomes after FMT or AKK administration should be interpreted as being associated with, rather than directly caused by, changes in HPA axis activity. Behavioral performance was evaluated at 1, 3, and 7 days after reperfusion. Longa neurological scores showed that both HFD^FMT^ and HFD^AKK^ groups had significantly lower scores than HFD^PBS^ controls, reflecting improved neurological recovery (Fig. 4f). This difference was evident on day 7, when Longa scores were reduced by 0.70 points in the HFD^FMT^ group and 0.90 points in the HFD^AKK^ group compared with HFD^PBS^ mice. In the rotarod test, latency to fall was significantly prolonged in the HFD^AKK^ group at all examined time points, indicating consistently enhanced motor coordination. In contrast, the HFD^FMT^ group exhibited a trend toward improvement during the early phase, which became statistically significant on day 7. At this time point, rotarod latency was increased by 63.3 s in HFD^FMT^ mice and 84.5 s in HFD^AKK^ mice compared with HFD^PBS^ controls (Fig. 4g). No significant differences were found between the HFD^AKK^ and HFD^FMT^ groups in either neurological scores or rotarod performance at any time point.

Open-field testing further supported these behavioral results. Representative trajectory plots and heatmaps revealed greater spontaneous locomotor activity in both intervention groups compared with HFD^PBS^ controls. Quantitative analysis showed that total movement distance was significantly greater in the HFD^AKK^ group at all time points after MCAO, whereas the HFD^FMT^ group showed increased locomotion with a statistically significant difference on day 7 (Fig. 4h and i). On day 7, total movement distance was approximately doubled in the HFD^FMT^ group and increased by more than twofold in the HFD^AKK^ group relative to HFD^PBS^ controls.

Together, these findings suggest that FMT and AKK administration were associated with improved post-stroke functional recovery and partial restoration of HPA axis hormone profiles in HFD mice, with AKK showing relatively earlier and more consistent behavioral effects.

### Gut microbiota intervention regulates hypothalamic CRH expression and correlates with reduced neuroinflammation and neuronal apoptosis after ischemic stroke

**Fig 5:**
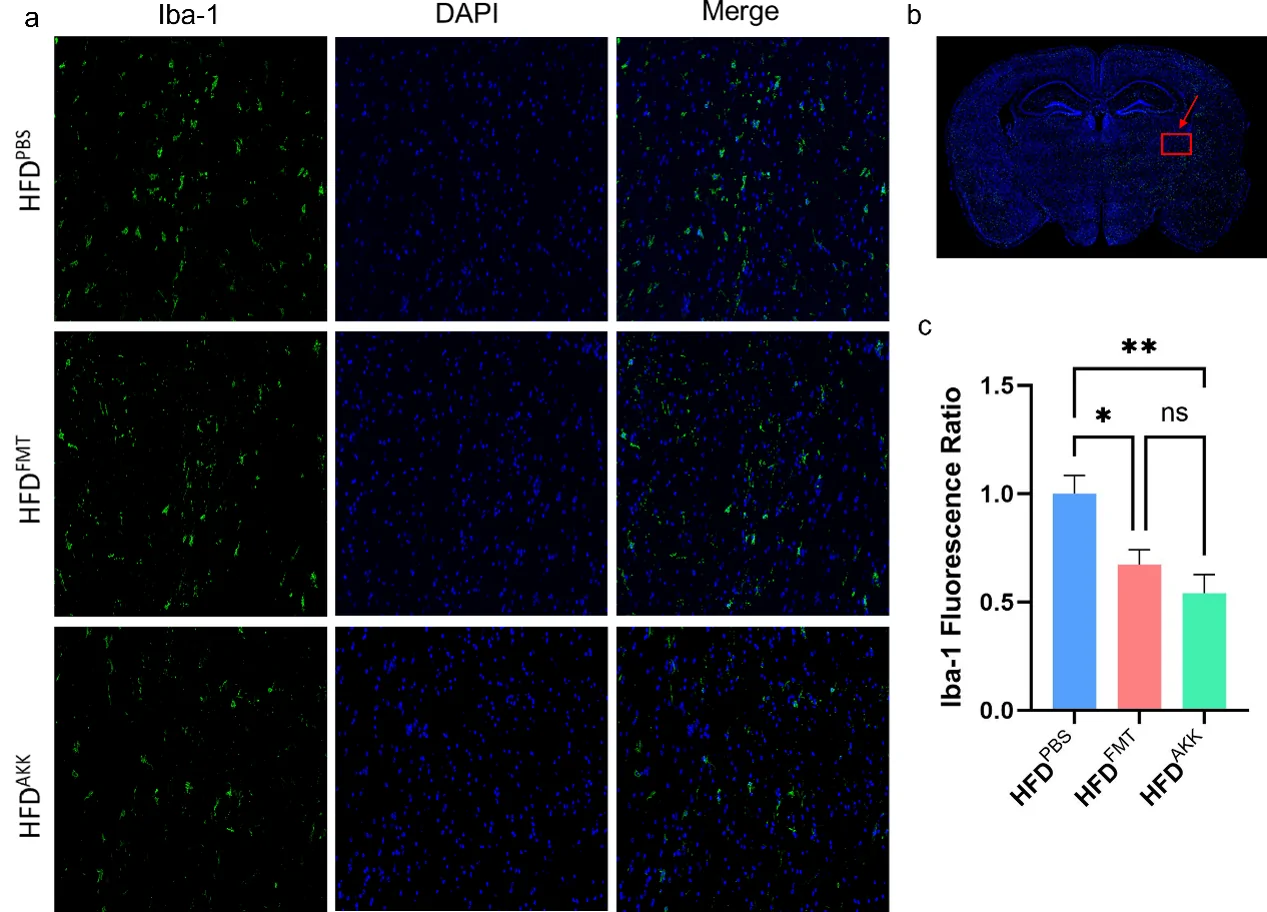
Gut microbiota intervention alleviates neuroinflammation following MCAO. (**a**) Representative immunofluorescence staining of Iba-1 (green) in the hypothalamic region across groups. (**b**) Analysis region for microglial activation was defined as the peri-infarct area. (**c**) Normalized quantification of mean fluorescence intensity of Iba-1 in each group, with the HFD^PBS^ group used as the control. *n* = 5 per group. *, *P* < 0.05; **, *P* < 0.01; ***, *P* < 0.001; ns, not significant.

To further investigate the effects of gut microbiota intervention on MCAO outcomes, we performed immunofluorescence staining on brain tissues collected from some mice on day 1 post-MCAO. First, consistent with the peripheral hormone results, hypothalamic CRH immunofluorescence further showed increased CRH immunoreactivity after FMT and AKK administration, supporting partial restoration of central HPA axis activity in HFD mice (Fig. S1a through c). These findings suggest that gut microbiota may regulate HPA axis activity by modulating hypothalamic CRH secretion. Next, we examined neuroinflammatory responses in the brain. Microglial activation was assessed via Iba-1 immunostaining. Results showed that microglial infiltration in the peri-infarct region was markedly reduced in both the HFD^FMT^ and HFD^AKK^ groups compared to the HFD^PBS^ group, with the HFD^AKK^ group demonstrating a more notable reduction. However, the difference between the HFD^AKK^ and HFD^FMT^ groups was not statistically significant (Fig. 5a through c). These findings indicate that gut microbiota intervention attenuates post-stroke neuroinflammation. Furthermore, TUNEL staining was performed to assess neuronal apoptosis within the infarct area. Compared to the HFD^PBS^ group, the number of TUNEL-positive cells was significantly reduced in the HFD^AKK^ group, whereas the reduction in the HFD^FMT^ group did not reach statistical significance (*P* = 0.0574). These findings suggest that AKK administration may mitigate post-ischemic neuronal apoptosis, whereas the potential anti-apoptotic effect of FMT requires further confirmation (Fig. S2a through c).

### Gut microbiota intervention remodels gut microbiome with characteristic enrichment of *Akkermansia*

**Fig 6:**
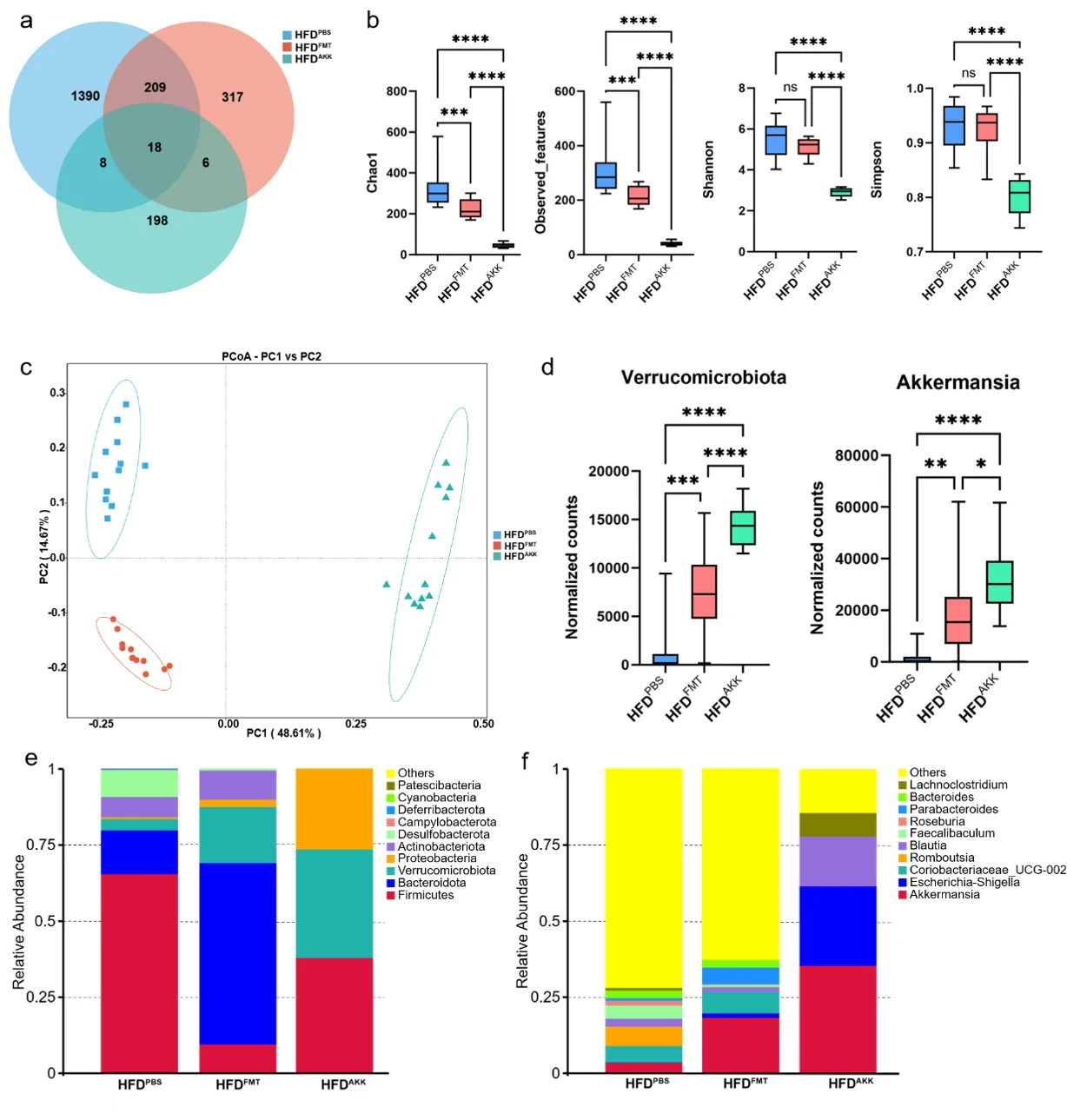
Gut microbiota restructuring following fecal microbiota transplantation and AKK administration.(**a**) Venn diagram showing shared and unique ASVs among the HFD^PBS^, HFD^FMT^, and HFD^AKK^ groups. (**b**) Alpha-diversity indices, including Chao1, observed features, Shannon, and Simpson, across the three groups. (**c**) PCoA showing separation of gut microbial communities among groups. (**d**) Normalized counts of *Verrucomicrobiota* and *Akkermansia* in each group. (**e and f**) Stacked bar plots showing relative microbial composition at the phylum and genus levels. *n* = 12 per group. ns, not significant; *P < 0.05; **P < 0.01; ***P < 0.001; ****P < 0.0001.

To assess alterations in gut microbiota following fecal microbiota transplantation and *Akkermansia* administration, fecal samples were collected 1 day prior to MCAO (24 h post-final gavage) and analyzed via 16S rRNA amplicon sequencing. Venn diagram analysis identified 18 shared ASVs across HFD^PBS^, HFD^FMT^, and HFD^AKK^ groups, with unique ASVs numbering 1,390 (HFD^PBS^), 317 (HFD^FMT^), and 198 (HFD^AKK^) (Fig. 6a). Alpha diversity analysis revealed reduced microbial richness in HFD^FMT^ and HFD^AKK^ groups compared to HFD^PBS^ controls (Fig. 6b). Beta diversity further demonstrated significant compositional differences among all groups (Fig. 6c).

Taxonomically, at the phylum level, HFD^FMT^ mice exhibited increased relative abundances of *Verrucomicrobiota* and *Bacteroidota* versus HFD^PBS^, resembling the normal diet profile, while the HFD^AKK^ group showed more pronounced *Verrucomicrobiota* expansion alongside elevated *Proteobacteria* and reduced *Firmicutes* (Fig. 6d and e). Genus-level analysis confirmed significantly higher *Akkermansia* abundance in both HFD^FMT^ and HFD^AKK^ groups (Fig. 6d and f). These interventions thus restructured the HFD-disrupted microbiota, characterized by *Akkermansia* enrichment but reduced overall microbial diversity.

## DISCUSSION

In this study, we investigated the role of gut microbiota in modulating stroke outcomes in mice fed a high-fat diet, with a focus on the HPA axis as a potential intermediary. Our results demonstrate that 8 weeks of HFD feeding significantly worsened post-ischemic neurological deficits, increased microglial activation and neuronal apoptosis, and suppressed HPA axis activity, as reflected by reduced CRH, ACTH, and corticosterone levels. These findings are consistent with previous reports that metabolic disturbances induced by chronic HFD can impair neuroendocrine function and exacerbate cerebral injury following stroke (14, 28–30). More importantly, our findings show that FMT and AKK administration were associated with partial restoration of hypothalamic CRH immunoreactivity, increased circulating HPA axis hormone levels, and improved post-stroke functional outcomes in HFD mice. However, these data do not establish a causal relationship between HPA axis changes and functional recovery. These observations suggest that microbial modulation of the HPA axis may represent a viable therapeutic strategy for ischemic stroke in the context of metabolic dysfunction.

While our data demonstrate that gut microbiota-targeted interventions can elevate CRH and coincide with improved stroke outcomes in HFD-fed mice, it remains unclear whether these effects are causally mediated by hormonal restoration. The observed associations, although statistically significant, do not confirm direct endocrine causality. Notably, alpha diversity was reduced after FMT and AKK administration compared with HFD^PBS^ mice, despite the beneficial effects of these interventions on hormone levels and post-stroke outcomes. Therefore, these findings should not be interpreted as evidence that microbiota intervention improved recovery by restoring overall microbial diversity. Instead, the protective effects may be more closely related to specific compositional or functional changes, such as *Akkermansia*-associated alterations and modulation of gut–brain neuroendocrine signaling. Prior evidence supports the notion that microbial components and metabolites can influence HPA axis regulation, although these intermediate pathways were not directly assessed in the present study (31, 32). For instance, *Akkermansia muciniphila* has been shown to enhance gut barrier integrity, reduce systemic endotoxemia, and suppress pro-inflammatory TLR4/NF-κB signaling cascades—mechanisms that may indirectly modulate hypothalamic CRH neuron activity and dampen neuroinflammation (33, 34). Moreover, in chronic stress models, *Akkermansia* administration restored HPA axis responsiveness and alleviated behavioral deficits, further implicating a gut-endocrine link (35, 36). We speculate that specific microbial taxa, such as *Akkermansia*, may serve not only as anti-inflammatory agents but also as modulators of central neuroendocrine circuits. Nonetheless, targeted mechanistic studies using CRH neuron-specific tracing, microbial metabolite profiling, or vagotomy models are needed to delineate the precise pathways through which gut microbiota influence the HPA axis and stroke outcomes.

Beyond neuroendocrine modulation, gut microbiota may also exert protective effects through endocrine-independent mechanisms, particularly by attenuating neuroinflammation and neuronal apoptosis (37–39). Both FMT and *Akkermansia* administration were associated with reduced microglial activation and fewer TUNEL-positive neurons in the peri-infarct cortex, suggesting attenuation of post-stroke neuroinflammation and apoptosis. While glucocorticoids are known anti-inflammatory agents, the degree of immune modulation observed in our study may not be fully explained by the modest elevations in corticosterone alone. Given the modest changes in circulating corticosterone and the pronounced reduction in inflammatory and apoptotic markers, non-HPA mechanisms may account for a substantial portion of the observed benefit after FMT or AKK administration. Indeed, the microbiota can influence systemic and central immune responses through multiple parallel mechanisms, including modulation of cytokine networks, microglial priming, and monocyte trafficking (40–43). It is possible that *Akkermansia*-mediated changes in gut permeability or immune cell profiles contribute independently to neuroprotection after stroke. These alternative pathways warrant further investigation, as they may operate synergistically or independently from HPA axis effects.

Our microbiome profiling results further support the notion that both FMT and *Akkermansia* administration reshaped the gut microbial ecosystem in a manner distinct from the HFD baseline. While alpha diversity was reduced in the intervention groups—perhaps due to the selective colonization of dominant taxa—β diversity analyses indicated a clear shift in overall composition. This reduced diversity may reflect dominance of selected taxa, including *Proteobacteria*, and its potential long-term consequences require cautious interpretation. Notably, *Akkermansia* abundance was significantly increased in both the AKK and FMT groups, suggesting successful colonization and potential functional relevance. At the phylum level, FMT more closely resembled the normal diet microbial structure, while *Akkermansia* enrichment led to a unique microbial signature characterized by elevated *Verrucomicrobiota* and *Proteobacteria*. These distinct compositional changes raise the possibility that microbial community structure, not merely hormone levels, may determine host physiological outcomes. Future studies using gnotobiotic models, defined microbial consortia, metabolomics, and targeted SCFA or bile acid analyses will be needed to identify the specific microbial metabolites and pathways involved. In addition, the increased relative abundance of *Proteobacteria* in the HFD^AKK^ group should be interpreted cautiously, as expansion of this phylum is often associated with inflammatory or dysbiotic states. Although AKK administration improved behavioral and neuroendocrine outcomes in the present study, this compositional change indicates that its microbiota-level effects are complex and not uniformly beneficial.

This study also raises broader questions regarding patient stratification and therapeutic translation. Our results imply that individuals with suppressed HPA axis activity, such as those with metabolic syndrome or obesity, may be more susceptible to poor stroke outcomes and may benefit from microbiota-based interventions. In contrast, patients with normal or elevated glucocorticoid levels may not derive the same benefit and could even be harmed by additional endocrine stimulation. However, this clinical implication remains speculative. HPA axis suppression in obese or metabolic syndrome stroke patients has not yet been systematically linked to specific gut microbiota signatures, and substantial clinical validation will be needed before translation. Emerging concepts in stroke medicine increasingly advocate for stratified interventions (44, 45). For instance, gut microbial signatures, such as high *Firmicutes/Bacteroidetes* ratio or depleted short-chain fatty acids (SCFA) producers, may serve as surrogate biomarkers for identifying patients likely to benefit from microbiota-based therapies (46–49). Integration of gut-endocrine-inflammation axes into stroke prognostication models could enable more refined, personalized interventions (50).

### Limitations

Several limitations of this study should be acknowledged. First, only male mice were used. It is well recognized that males and females differ in hormonal regulation, particularly in HPA axis reactivity and in the composition and functional diversity of the gut microbiota. Because ischemic stroke affects both sexes, it remains uncertain whether the gut–HPA–stroke associations identified here are equally applicable to females. Moreover, *Akkermansia*–HPA axis interactions may be sex-specific, which further limits the generalizability of the present male-only findings. The exclusive use of male animals in this study was intended to minimize the confounding influence of estrous cycle–related hormonal fluctuations on endocrine and behavioral measurements. Nevertheless, future studies should include both male and female subjects to determine whether sex-specific hormonal responses or microbial compositions alter the effects of microbiota-targeted interventions.

Second, both FMT and AKK treatment were administered before MCAO as preventive interventions. While this design enabled us to explore how microbial modulation influences pre-stroke endocrine status and subsequent outcomes, it does not fully recapitulate the clinical context, where therapeutic interventions typically occur after stroke onset. Therefore, the preventive nature of our design, together with the relatively short 7-day pretreatment window, limits its direct translational applicability and may not fully model long-standing microbiome modulation. Besides, the reduction in body weight after FMT or AKK administration represents a potential confounding factor, as improved neurological outcomes may partly reflect weight loss or broader metabolic improvement. Future studies including weight-matched controls and more comprehensive metabolic measurements will be needed to disentangle microbiota–HPA axis effects from general metabolic effects. The AKK dose was selected based on a previous study, but dose–response effects were not examined; therefore, the dose may not represent the optimal therapeutic dose, and potential under- or overdosing cannot be excluded. Future studies should evaluate post-stroke treatment paradigms to determine the therapeutic potential and optimal intervention window of microbiota-based strategies.

In addition, the mechanistic relationship between gut microbial alterations, CRH regulation, and corticosterone secretion remains incompletely understood and requires further confirmation using direct approaches, such as HPA axis blockade or targeted modulation of CRH signaling. Infarct volume was not directly quantified, limiting anatomical interpretation of the behavioral outcomes. Metabolic phenotyping was also limited to body weight, without glucose, insulin, lipid, or other metabolic endpoints, limiting the interpretation of metabolic changes after intervention. Moreover, recipient mice did not receive antibiotic pretreatment before FMT, which may have affected donor microbiota engraftment. Further research integrating infarct-volume assessment, metabolic evaluation, and causal manipulation approaches is required to clarify whether microbiota-induced hormonal changes directly mediate neuroprotection after stroke.

### Conclusion

In summary, this study demonstrates that a high-fat diet disrupts gut microbiota composition and HPA axis function, which together contribute to aggravated neurological deficits after ischemic stroke. Restoration of microbial balance through FMT or AKK supplementation elevated circulating HPA axis hormones and improved post-stroke behavioral outcomes in HFD-fed mice. Among the two interventions, AKK treatment produced more pronounced benefits in both endocrine and functional recovery. These findings provide new evidence linking diet-induced gut dysbiosis to neuroendocrine dysfunction and stroke vulnerability, emphasizing the importance of the gut–HPA–brain axis in mediating metabolic influences on brain injury. Although the causal relationship between hormonal restoration and functional improvement remains to be fully clarified, this work lays a foundation for future studies exploring microbiota-based strategies for protecting the brain under metabolic stress conditions.

## Supplementary Material

Reviewer comments

## Data Availability

The data supporting the findings of this study are publicly available in Mendeley Data at DOI: 10.17632/8228hrsssb.1.

## SUPPLEMENTAL MATERIAL

Fig. S1 and S2 spectrum.03549-25-s0001.docx Figure S1: Gut microbiota intervention regulates hypothalamic CRH expression. Figure S2: Gut microbiota intervention alleviates neuronal apoptosis after MCAO.

OPEN PEER REVIEW reviewer-comments.pdf An accounting of the reviewer comments and feedback.

ASM does not own the copyrights to Supplemental Material that may be linked to, or accessed through, an article. The authors have granted ASM a non-exclusive, world-wide license to publish the Supplemental Material files. Please contact the corresponding author directly for reuse.
