# Supplementary material for: Gut dysbiosis impairs HPA axis function and exacerbates post-stroke inflammation in high-fat diet-fed male mice
Source: Microbiol Spectr. 2026 Aug 21;14(10):e03549-25. doi: 10.1128/spectrum.03549-25 (PMC13689323; doi:10.1128/spectrum.03549-25)
Supplement: Supplementary file 1 [file reviewer-comments.pdf]

ASM | Microbiology Spectrum

Gut Dysbiosis Impairs HPA Axis Function and Exacerbates Post-Stroke Inflammation in High-Fat Diet-Fed Male Mice

Hanlin Zhong, Zeping Jin, Yiqi Liu, Yang Wang, and Yunpeng Liu

Corresponding Author(s): Yunpeng Liu, Beijing Chaoyang Hospital Affiliated to Capital Medical University

Review Timeline:

Submission Date:	November 4, 2025
Editorial Decision:	May 7, 2026
Revision Received:	July 4, 2026
Editorial Decision:	August 2, 2026
Revision Received:	August 3, 2026
Accepted:	August 5, 2026

Editor: Wanilada Rungrassamee

Reviewer(s): Disclosure of reviewer identity is with reference to reviewer comments included in decision letter(s). The following individuals involved in review of your submission have agreed to reveal their identity: Imran Hossain (Reviewer #1)

Transaction Report:

(Note: With the exception of the correction of typographical or spelling errors that could be a source of ambiguity, letters and reports are not edited. The original formatting of letters and referee reports may not be reflected in this compilation.)

DOI: https://doi.org/10.1128/spectrum.03549-25

Re: Spectrum03549-25 (**Gut Dysbiosis Impairs HPA Axis Function and Exacerbates Post-Stroke Inflammation in High-Fat Diet-Fed Male Mice**)

Dear Dr. Yunpeng Liu:

Thank you for the privilege of reviewing your work. Below you will find instructions from the Spectrum editorial office, and the reviewer comments.

Please return the manuscript within 60 days; if you cannot complete the modification within this time period, please contact me. If you do not wish to modify the manuscript and prefer to submit it to another journal, notify me immediately so that the manuscript may be formally withdrawn from consideration by Spectrum.

Revision Guidelines
To submit your modified manuscript, log into the submission site at https://spectrum.msubmit.net/cgi-bin/main.plex. Go to Author Tasks and click the appropriate manuscript title to begin. The information you entered when you first submitted the paper will be displayed; update this as necessary. Note the following requirements:

• Upload point-by-point responses to the issues raised by the reviewers in a file named "Response to Reviewers," NOT in your cover letter.
• Upload a compare copy of the manuscript (without figures) as a "Marked-Up Manuscript" file.
• Upload a clean .DOC/.DOCX version of the revised manuscript and remove the previous version.
• Each figure must be uploaded as a separate, editable, high-resolution file (TIFF or EPS preferred), and any multipanel figures must be assembled into one file.
• Any supplemental material intended for posting by ASM should be uploaded with their legends separate from the main manuscript. You can combine all supplemental material into one file (preferred) or split it into a maximum of 10 files with all associated legends included.

For complete guidelines on revision requirements, see our Submission and Review Process webpage. Submission of a paper that does not conform to guidelines may delay acceptance of your manuscript.

Data availability: ASM policy requires that data be available to the public upon online posting of the article, so please verify all links to sequence records, if present, and make sure that each number retrieves the full record of the data. If a new accession number is not linked or a link is broken, provide Spectrum production staff with the correct URL for the record. If the accession numbers for new data are not publicly accessible before the expected online posting of the article, publication may be delayed; please contact production staff (Spectrum@asmusa.org) immediately with the expected release date.

Publication Fees: For information on publication fees and which article types are subject to charges, visit our website. If your manuscript is accepted for publication and any fees apply, you will be contacted separately about payment during the production process; please follow the instructions in that e-mail. Arrangements for payment must be made before your article is published.

ASM Membership: Corresponding authors may join or renew ASM membership to obtain discounts on publication fees. Need to upgrade your membership level? Please contact Customer Service at Service@asmusa.org.

The ASM Journals program strives for constant improvement in our submission and publication process. Please tell us how we can improve your experience by taking this quick Author Survey.

Thank you for submitting your paper to Spectrum.

Sincerely,
Wanilada Rungrassamee
Editor
Microbiology Spectrum

Reviewer #1 (Public repository details (Required)):

The study includes 16S rRNA sequencing data

Reviewer #1 (Comments for the Author):

Global stylistic and structural comments:
The manuscript is generally well written, but there are several minor grammar and punctuation issues (e.g., inconsistent hyphenation, misuse of commas, double periods in lines 59-60, "gut-microbiota-HPA-axis axis" at line 16-17). A careful language edit would help.

The logic flow is good: Introduction → design → HFD effects → microbiota changes → interventions → mechanistic discussion. However, you sometimes repeat statements (e.g., benefits of Akkermansia) across sections; condensing these would improve readability.

You might consider adding a schematic summarizing putative mechanisms (gut dysbiosis → CRH/ACTH/corticosterone changes → microglial activation/apoptosis → behavioral outcome), clearly labeled as hypothetical.

Some of my suggestions:
1. Line 16-17: "gut-microbiota-HPA-axis axis" is redundant; revise to "gut-microbiota-HPA axis."
2. Lines 20-27: Importance section nicely frames translational relevance, but it essentially repeats the abstract. You could shorten by one or two sentences to avoid redundancy and emphasize what is novel (HFD context, CRH suppression, Akkermansia as a candidate).
3. Lines 36-39: "a definitive causal link between elevated hormone levels and poor prognosis has not been established" should be supported by more explicit reference to null results or conflicting data, not just . Right now, the logical contrast with the previous sentence is a bit weak.
4. Lines 52-59: You state that chronic HFD "has been shown to alter the activity of the HPA axis, disrupt glucocorticoid signaling, and potentiate neuroinflammatory cascades" but then say "some inconsistencies remain regarding the direction and extent of these hormonal changes." It would help to very briefly (1-2 clauses) clarify which studies show increased vs decreased HPA activity to pre-emptively address the apparent inconsistency.
5. Lines 60-62: Use of "--" is nonstandard; replace with a single long dash ("-") or standard punctuation. Look like AI writing.
6. Lines 69-72: The knowledge gap is well-stated ("direct experimental evidence delineating how HFD-induced gut microbiota alterations impact the HPA axis and stroke outcomes remains limited"), but the last sentence of the paragraph slightly overpromises; make clear that you will explore association and partial mechanistic inference rather than directly proving causality.
7. Lines 75-82: The Akkermansia rationale is strong but somewhat scattered. Consider tightening to one compact argument: metabolic benefits, barrier integrity, and prior evidence for HPA modulation, then lead directly into your hypothesis.
8. Lines 84-86: "precise, controllable, and low-risk intervention strategy" feels overstated for a preclinical mouse study. Consider softening to "a potentially precise and controllable adjunctive strategy warranting further preclinical and clinical evaluation."
9. Lines 89-101: The two-part design is clear, but you never explicitly state whether there is an HFD group without stroke in the first part for baseline HPA measurements or microbiota assessment. Clarify which groups exist in each part and whether there are non-MCAO controls.
10. Lines 101-110: Group sizes are given (n=10 behavioral, n=10 histology per group), but it is not clear if this is per cohort or total across experiments. Clarify whether experiments were performed once or repeated and pooled.
11. Lines 109-110: You state "12 mice per group were randomly selected for 16S rRNA sequencing." Clarify whether these 12 are from the same pool as the n=20 per group, and if so, how overlap with behavioral or histology subgroups was handled (e.g., did some animals contribute both behavior and microbiome data?).
12. Lines 111-120: Sex is limited to male mice, which you acknowledge later in the Limitations. It may be worth pre-emptively mentioning here that only males were used and that rationale will be discussed.
13. Lines 119-120: You state that allocation and assessment were blinded, but you do not describe the randomization procedure (e.g., block randomization, random number generator) or whether cage effects were controlled. Adding a brief description would strengthen rigor.
14. Lines 122-131: Criteria for successful MCAO is "reduction in cerebral blood flow to below 60% of the baseline level, maintained for 60 minutes." You do not state whether animals failing to meet this criterion were excluded and, if so, how many. A CONSORT-style statement on exclusions is important (e.g., number excluded for surgical reasons, death, failure in flow reduction).
15. Lines 132-137: You measure CRH in plasma, which is unconventional and technically challenging; it is important to specify (1) whether this is serum or plasma, (2) validation of the CRH assay in mouse samples (e.g., detection limit, cross-reactivity), and (3) whether samples were run in duplicate/triplicate and intra/inter-assay CV. As CRH is a central mechanistic endpoint, analytical reliability is critical.
16. Lines 155-161: You describe collection and sequencing platform, but important details are missing:
Which 16S region (e.g., V3-V4) and primer set were used?
How were reads processed (e.g., DADA2, QIIME2)?
Which reference database and taxonomy classifier?
Were rarefaction depths standardized across samples?
Without these information, reproducibility is limited.
17. Lines 163-177, 179-188: Methods are fairly detailed, but you do not describe quantification procedures (e.g., how many sections per animal, how many fields per section, selection criteria, and whether thresholding/analysis were done blinded). These details should be stated here, not only in figure legends.
18. Lines 190-199: FMT protocol is standard; however you do not mention whether recipient mice received antibiotics prior to

FMT, which is common to facilitate colonization. This omission is important because colonization efficiency and interpretation of microbiota changes differ with/without antibiotic preconditioning.
19. Lines 201-209: You justify AKK dose with a previous study but explicitly state that "The dose-response relationship was not examined in the present study." This should be acknowledged again as a limitation later (dose not optimized, potential under/overdosing).

Results
3.1 HFD, outcomes, and HPA axis (Lines 223-246)
Lines 227-234: You describe body weight and behavioral deficits; however, absolute body weight values, infarct size, and baseline performance are not reported in text. Consider including key numbers (e.g., mean weight {plus minus} SEM, Longa scores) to give the reader a sense of effect size.

Lines 237-239: "HFD mice exhibited significantly lower levels of corticosterone, ACTH, and CRH levels at all time points compared to normal diet controls" is strong; indicate whether baseline pre-MCAO hormone levels already differed (they did by design, but emphasize this) and whether there is any time × diet interaction. A simple "at all time points" hides potential differences in dynamic response vs baseline shift.

Lines 241-243: Correlation R^2 values (0.35 and 0.22) show modest relationships; mention that correlations, while significant, explain only a portion of variance. Avoid overinterpreting as strong predictive relationships.

3.2 CRH, microglia, apoptosis (Lines 259-278)
Lines 261-264: You conclude that reduced hypothalamic CRH expression "suggests" impaired HPA output. This is plausible but you do not distinguish between CRH neuron number loss vs reduced expression per neuron. Clarify in the text what exactly was quantified (cell counts vs mean fluorescence intensity) and whether region boundaries were precisely defined.

Lines 269-275: Microglial and TUNEL data are described qualitatively but again without numerical values or effect sizes. Adding the magnitude of changes (% increase in Iba-1 intensity, fold change in TUNEL+ cells) would help readers assess biological relevance.

3.3 HFD and microbiota (Lines 288-305)
Lines 291-297: You show that HFD reduces alpha diversity and alters beta diversity. It would be useful to explicitly state which diversity indices show significant differences (Chao1 and observed features are mentioned, but p-values are not given in text).

Lines 299-301: The phylum-level shifts (increased Firmicutes, Actinobacteria, Desulfobacterota; decreased Verrucomicrobiota, Lactobacillota) are described but without mentioning statistical test or correction; clarify that these differences are statistically significant and indicate whether you controlled for multiple testing.

Lines 302-304: Akkermansia reduction is central to the intervention concept; consider quantifying its relative abundance (e.g., median %, IQR) to emphasize the magnitude of depletion.

3.4 Microbiota interventions and functional recovery (Lines 314-352)
Lines 325-325: HFD mice receiving FMT or AKK had reduced body weight compared with HFDPBS. This is important because it introduces a potential confounder: improved outcomes may partially reflect weight loss/metabolic improvement rather than solely microbiota-HPA effects. You should acknowledge this in the Results (not only Discussion) and ideally adjust or at least discuss in terms of metabolic parameters.

Lines 327-333: You state that ACTH and corticosterone are significantly elevated, whereas CRH increases but does not reach significance in HFDFMT. This suggests that CRH might not be the primary driver of peripheral hormone changes, or that power is limited; you should note this nuance explicitly and avoid presenting CRH restoration as equally robust in all groups later.

Lines 335-342: Longa and rotarod improvements are described, with AKK showing earlier, stronger effects than FMT. It would help to state effect sizes (e.g., mean difference in Longa score vs HFDPBS on day 7) and mention whether there were interactions between time and treatment (since improvements emerge over days).

Lines 349-352: You state "Although elevated HPA-axis hormones accompanied functional improvement, the present data do not establish a causal relationship." This is an important caveat and might be moved forward in the paragraph to prevent readers from inferring causality earlier.

3.5 CRH, microglia, apoptosis after intervention (Lines 367-387)
Lines 371-376: You refer to "Figure S1a-c" for CRH restoration after FMT and AKK, but these are supplementary figures; given that CRH is a central mechanistic link, consider moving these data into main figures or at least referencing them more strongly.

Lines 378-383: Iba-1 and TUNEL changes are presented, but the phrase "more notable reduction" and "a decreasing trend ($p = 0.0574$)" risks overemphasizing near-significant results. It is better to state clearly that TUNEL reduction in HFDFMT did not

reach significance and treat it as hypothesis-generating rather than quasi-positive.

3.6 Microbiota restructuring (Lines 394-416)
Lines 399-402: Alpha diversity is reduced in intervention groups compared with HFDPBS. This is a paradoxical finding given that interventions are beneficial; you should highlight this tension more clearly and avoid implying that diversity restoration is the mechanism of benefit.

Lines 404-406: The phylum-level differences in HFDAKK (higher Proteobacteria) might raise concerns about potential pro-inflammatory consequences; this deserves explicit mention and cautious interpretation.

Discussion and Limitations
Interpretation and mechanistic claims
Lines 420-429: The summary of findings is accurate but somewhat overstated in mechanistic certainty ("interventions...partially restored CRH expression..., elevated systemic glucocorticoid levels, and improved...outcomes"). You should reiterate that causality is not demonstrated and that HPA changes may be one of several mediating pathways.

Lines 435-446: You nicely acknowledge that association does not prove endocrine causality. However, later sentences (e.g., lines 441-443) risk reintroducing a causal tone ("may serve...as modulators of central neuroendocrine circuits") without clearly framing it as speculative. Consider adding explicit wording like "We speculate that..." or "It is plausible but remains unproven that...".

Lines 448-459: The discussion of endocrine-independent pathways is strong, but you may want to explicitly highlight that, given modest corticosterone changes and strong anti-inflammatory effects, non-HPA mechanisms may account for a substantial portion of benefit.

Microbiome interpretation
Lines 462-469: You discuss reduced alpha diversity in interventions but do not critically reflect on whether this may carry risks or reflect dominance of a few taxa (including Proteobacteria). Adding a short caveat would demonstrate balanced interpretation.

Lines 471-472: Suggesting gnotobiotic and defined consortia approaches is appropriate; you might also suggest metabolomics and targeted SCFA or bile acid analysis as next steps.

Clinical translation and stratification
Lines 474-482: The idea that patients with suppressed HPA axis may benefit from microbiota-based approaches is interesting but highly speculative at this stage. You should explicitly say that clinical HPA suppression in obese/MetS stroke patients has not yet been systematically linked to microbiota signatures in the way your model suggests, and that translation will require substantial validation.

Lines 478-481: The sentence beginning "Moreover, Emerging concepts in stroke medicine..." has capitalization and stylistic issues ("Emerging" should not be capitalized; "Moreover," is unnecessary).

Limitations section
Lines 486-495: The sex limitation is well discussed, but you might also highlight that Akkermansia-HPA interactions could be sex-specific, making male-only data particularly hard to generalize.

Lines 497-502: You acknowledge the preventive design; this is appropriate. You might also mention that the 7-day pre-treatment window is relatively short and does not model long-standing microbiome modulation.

Lines 504-507: You mention that mechanistic relationships remain incompletely understood; this could be strengthened by explicitly acknowledging the absence of HPA blockade experiments (e.g., glucocorticoid receptor antagonists, adrenalectomy) and lack of direct CRH neuron manipulation.

Missing limitation:

No metabolic phenotyping beyond body weight (e.g., glucose, insulin, lipids) is provided, limiting interpretation of how "metabolic stress" was modulated by interventions. Adding a note about missing metabolic endpoints would strengthen transparency.

Antibiotics not used before FMT (if true) could be explicitly acknowledged as a factor that may limit colonization efficiency and generalizability to standard FMT protocols.

Reviewer #2 (Public repository details (Required)):

Gut microbiome data need to be deposited.

Reviewer #2 (Comments for the Author):

Manuscript title: Gut Dysbiosis Impairs HPA Axis Function and Exacerbates Post-Stroke Inflammation in High-Fat Diet-Fed Male Mice

Comments

1. The current conclusions, particularly those regarding therapeutic implications for stroke, are overconcluded given that the study relies exclusively on male data. Biological sex is a critical variable in stroke pathophysiology and treatment response; therefore, the results cannot be assumed to apply to female cohorts without direct investigation. The study design was only based on male mice therefore therapeutic implications for stroke patients cannot legitimately apply to female patients on the basis of male-only data. The abstract , discussion and throughout the draft need a revision to explicitly limit all conclusions to male animals.

2. The findings in this study were mainly correlational not a direct causation. I suggest the authors to revise the title, abstract, and discussion to use more conservative, association-based language (e.g., "is associated with" or "correlates with") rather than strongly address a direct cause, which could lead to scientifically misleading.

3. The data shown in the Result section were based only on behavioural scores (Longa, rotarod, open field) and inflammatory markers without establishing whether these differences are accompanied by proportional differences in infarct size. If HFD mice have larger infarcts, some or all of the behavioural deficit could be explained by greater tissue loss rather than impaired recovery. Could the authors add infarct volume as percentage of ipsilateral hemisphere to strengthen this observation?

4. In this study, the authors proposed that gut dysbiosis suppresses CRH through gut-barrier dysfunction and TLR4/NF-κB signalling, or via microbial metabolites, yet none of these intermediate steps were measured. Please elaborate on this matter or revise the draft to conclude only evidence supported by the data.

5. Iba-1 fluorescence intensity is useful as a proxy for microglial morphological activation. It could not distinguish pro- from anti-inflammatory states (M1/M2 polarisation), or capture infiltrating monocytes or other immune effectors. Therefore, the mentioned exacerbated neuroinflammation was derived based on a single histological marker without systemic inflamatory data. Could the authors further explain or clarify this conclusion?

6. The main key from this study was that microbiota intervention could restore HPA axis activity and that this hormonal restoration could improved stroke outcomes. This is a bit contradicted by the FMT data. Figure 4e shows that CRH levels in the HFD-FMT group do not significantly differ from HFD-PBS controls ($p > 0.05$; described as a "trend"). Yet the same group shows significantly improved Longa scores, rotarod performance, and open-field distance (Figs. 4f-i) and reduced Iba-1 staining (Fig. 5). If CRH restoration is the mechanism, how did a group without significant CRH elevation achieve equivalent behavioural benefit? Perhaps, the authors can discuss this discrepancy.

7. A single dose of 2×10^9 CFU/200 μL was used throughout, justified by reference to one prior study with a different disease model. Please elaborate more or add rationale why this single dose was selected.

Specific comments
1. Methods section (lines 163-178) only showed antibody concentrations and excitation/emission wavelengths but omits instrument settings critical for reproducibility of fluorescence intensity comparisons. Please add more details.

2. Lines 129-131 state the criterion but lines 102-103 present group sizes without accounting for surgical failures. I suggest the authors adding a statement in Methods or Results reporting the number of animals per group that met the CBF success criterion, the number excluded, and the total enrolled. Report whether CBF monitoring was continuous or sampled.

3. Need bioinformatics analysis details (Line 159-161).

4. For corticosterone assay, please add a time of day for blood collection of each group.

Response to Reviewers

Manuscript ID: Spectrum03549-25R1

Title: Gut Dysbiosis Impairs HPA Axis Function and Exacerbates Post-Stroke Inflammation in High-Fat Diet-Fed Male Mice

We sincerely thank the reviewers for their careful evaluation of our manuscript and for the constructive comments. We have revised the manuscript substantially to improve clarity, methodological transparency, and interpretive balance. In particular, we have softened causal and translational claims throughout the manuscript, clarified that the findings are based on male mice only, added methodological details for MCAO inclusion criteria, hormone assays, 16S rRNA sequencing, immunofluorescence quantification, and statistical analyses, and expanded the Limitations section to acknowledge the preventive design, lack of infarct-volume measurement, absence of direct HPA-axis manipulation, and limited metabolic phenotyping. All changes have been incorporated into the revised manuscript. Below, we provide a point-by-point response.

Reviewer #1

Comment 1: Line 16-17: "gut-microbiota-HPA-axis axis" is redundant; revise to "gut-microbiota-HPA axis."

Response: We revised this phrase to “gut microbiota–HPA axis.”

Revision:

The phrase has been corrected in the Abstract Line 17.

Comment 2: Lines 20-27: Importance section nicely frames translational relevance, but it essentially repeats the abstract. You could shorten by one or two sentences to avoid redundancy and emphasize what is novel (HFD context, CRH suppression, Akkermansia as a candidate).

Response:
Thank you for this helpful suggestion. We agree that the original Importance section partly repeated the Abstract. We have shortened this section and revised it to emphasize the novel aspects of the study, namely the high-fat diet context, the association between HFD-induced gut dysbiosis and suppressed HPA-axis activity, and *Akkermansia muciniphila* as a candidate microbiota-based intervention. Redundant statements regarding general stroke burden and broad translational implications have been removed.

Revision:

The Importance section has been revised as follows（Lines 21-28）:

High-fat diet-associated metabolic stress is known to worsen stroke outcomes, but how diet-induced gut dysbiosis interacts with neuroendocrine regulation after stroke remains insufficiently understood. The novelty of this study lies in linking HFD-associated microbial disruption with suppressed hypothalamic CRH expression, reduced HPA-axis hormone levels, and aggravated post-stroke inflammation in male mice. We further identify Akkermansia muciniphila as a candidate microbiota-based intervention associated with partial restoration of HPA-axis activity and improved functional recovery after ischemic injury.

Comment 3: Lines 36-39: "a definitive causal link between elevated hormone levels and poor prognosis has not been established" should be supported by more explicit reference to null results or conflicting data, not just . Right now, the logical contrast with the previous sentence is a bit weak.

Response:

We have revised this sentence to more explicitly refer to the inconsistent clinical evidence from corticosteroid treatment studies, thereby clarifying the contrast between observational associations and causal interpretation.

Revision:

Lines 39-41

However, given the inconsistent clinical efficacy of corticosteroid treatment in acute ischemic stroke, a definitive causal link between elevated hormone levels and poor prognosis has not been established.

Comment 4: Lines 52-59: You state that chronic HFD "has been shown to alter the activity of the HPA axis, disrupt glucocorticoid signaling, and potentiate neuroinflammatory cascades" but then say "some inconsistencies remain regarding the direction and extent of these hormonal changes." It would help to very briefly (1-2 clauses) clarify which studies show increased vs decreased HPA activity to pre-emptively address the apparent inconsistency.

Response:

We have revised this sentence to briefly clarify the apparent inconsistency.

Revision:

Lines 59-62

Although some studies have reported increased basal or stress-induced glucocorticoid output after HFD exposure, others have observed blunted HPA-axis responsiveness or reduced

corticosterone levels under prolonged metabolic stress; therefore, inconsistencies remain regarding the direction and extent of these hormonal changes.

Comment 5: Lines 60-62: Use of "--" is nonstandard; replace with a single long dash ("-") or standard punctuation. Look like AI writing.

Response:
Response: Thank you for pointing this out. We have replaced the nonstandard double dashes with a single long dash and checked the manuscript for similar formatting issues.

Revision:
Line 63

Moreover, obesity and metabolic syndrome-key consequences of chronic HFD-have been increasingly recognized

Comment 6: Lines 69-72: The knowledge gap is well-stated ("direct experimental evidence delineating how HFD-induced gut microbiota alterations impact the HPA axis and stroke outcomes remains limited"), but the last sentence of the paragraph slightly overpromises; make clear that you will explore association and partial mechanistic inference rather than directly proving causality.

Response:
Thank you for this helpful suggestion. We agree that the original sentence could be interpreted as implying direct causality. We have revised the final sentence of this paragraph to clarify that the present study examines associations between microbiota-targeted interventions, HPA-axis activity, endogenous hormone levels, and post-stroke outcomes, and provides only partial mechanistic insight rather than direct causal proof.

Revision:
Lines 74-77

To address these gaps, **the present study was designed to examine whether microbiota-targeted interventions in HFD-fed mice are associated with changes in HPA-axis activity, endogenous hormone levels, and post-stroke outcomes, thereby providing partial mechanistic insight into the gut microbiota–HPA-axis–stroke relationship.**

Comment 7: Lines 75-82: The Akkermansia rationale is strong but somewhat scattered. Consider tightening to one compact argument: metabolic benefits, barrier integrity, and prior evidence for HPA modulation, then lead directly into your hypothesis.

Response:
Response: Thank you for this helpful suggestion. We have tightened the rationale for *Akkermansia muciniphila* into a more compact argument. The revised paragraph now first

summarizes its metabolic and gut-barrier benefits, then notes prior evidence for HPA-axis modulation, and finally leads directly to our hypothesis that *Akkermansia* may act as a candidate microbiota-based intervention under HFD conditions.

Revision:

Lines 77-82

Among gut microbes, ***Akkermansia muciniphila*, a mucin-degrading bacterium enriched in healthy individuals, has emerged as an important regulator of metabolic homeostasis and intestinal barrier integrity. Previous studies have shown that *Akkermansia* supplementation can ameliorate obesity-related metabolic dysfunction, reduce inflammation, and modulate gut–brain signaling, including HPA-axis responses under stress-related conditions. Therefore, we hypothesized that *Akkermansia* may serve as a candidate microbiota-based intervention associated with improved neuroendocrine balance and reduced stroke-related injury under high-fat dietary conditions.**

Comment 8: Lines 84-86: "precise, controllable, and low-risk intervention strategy" feels overstated for a preclinical mouse study. Consider softening to "a potentially precise and controllable adjunctive strategy warranting further preclinical and clinical evaluation."

Response:

We agree that the original wording overstated the translational implications of a preclinical mouse study. We have softened this statement and now describe microbiota modulation as a potentially precise and controllable adjunctive strategy that requires further preclinical and clinical evaluation.

Revision:

Lines 86-88

This work aims to deepen our mechanistic understanding of gut microbiota-neuroendocrine interactions and to offer a **potentially precise and controllable adjunctive strategy warranting further preclinical and clinical evaluation.**

Comment 9: Lines 89-101: The two-part design is clear, but you never explicitly state whether there is an HFD group without stroke in the first part for baseline HPA measurements or microbiota assessment. Clarify which groups exist in each part and whether there are non-MCAO controls.

Response:

Thank you for pointing this out. We have clarified in the revised Methods that no separate sham-operated group was included, because the primary aim of the study was to examine the effects of HFD and microbiota intervention under ischemic conditions. We also clarified that

baseline hormone and microbiota measurements were obtained before MCAO.

Revision:

Lines 94-96.

In the first part, ……and HPA axis activity. **Because the primary aim was to examine the effects of HFD and microbiota intervention under ischemic conditions, no separate sham-operated group was included.** Blood samples for hormone assays were collected before MCAO and at 0.5, 1, 2, 4, and 6 hours after occlusion to evaluate dynamic hormonal responses to ischemic stress.

Comment 10: Lines 101-110: Group sizes are given (n=10 behavioral, n=10 histology per group), but it is not clear if this is per cohort or total across experiments. Clarify whether experiments were performed once or repeated and pooled.

Response:

We agree. We have clarified that the reported sample sizes represent the final total number of animals included in each analysis, rather than numbers per repeated cohort. We also specified that behavioral and histology/biochemistry analyses were performed in separate subgroups of mice, with n=10 per group for each subgroup.

Revision:

Lines 104-107.

For each experimental group (ND, HFD, HFDPBS, HFDFMT, and HFDAKK), **behavioral and histology/biochemistry analyses were performed in separate subgroups of mice, with n=10 per group for each subgroup. The reported sample sizes represent the final total number of animals included in each analysis, rather than numbers per repeated cohort.**

Comment 11: Lines 109-110: You state "12 mice per group were randomly selected for 16S rRNA sequencing." Clarify whether these 12 are from the same pool as the n=20 per group, and if so, how overlap with behavioral or histology subgroups was handled (e.g., did some animals contribute both behavior and microbiome data?).

Response: Thank you for this important point. We have clarified how the 16S rRNA sequencing samples overlapped with the behavioral and histological/biochemical subgroups. Fecal samples were collected from all animals before MCAO, and 12 mice per group were randomly selected from the same experimental pool for microbiome analysis. Therefore, some animals contributed both microbiome data and behavioral or histological/biochemical data. Because fecal collection was performed before MCAO and did not interfere with later behavioral testing or tissue collection, this overlap was not expected to influence the outcome measurements. We have also clarified that the microbiome data were analyzed at the group

level and were not treated as an independent validation cohort or used to make animal-level causal inferences.

Revision:

Lines 114-116.

Fecal samples were collected one day before MCAO, and 12 mice per group were randomly selected for 16S rRNA sequencing to analyze gut microbiota composition. **Some animals therefore contributed both microbiome data and behavioral or histological/biochemical data, but fecal collection was performed before MCAO and did not interfere with subsequent outcome assessments.**

Comment 12: Lines 111-120: Sex is limited to male mice, which you acknowledge later in the Limitations. It may be worth pre-emptively mentioning here that only males were used and that rationale will be discussed.

Response:

Thank you for this helpful suggestion. We have added a sentence in the Animals and ethics statement section to explicitly state that only male mice were used, in order to reduce potential variability associated with estrous cycle-related hormonal fluctuations. We also indicate that the implications of this sex limitation are discussed in the Limitations section.

Revision:

Lines 119-121

Six-week-old C57BL/6J mice (18-22 g at baseline; Vital River Laboratory Animal Technology, Beijing, China) were used. **Only male mice were used in this study to reduce potential variability associated with estrous cycle-related hormonal fluctuations; the implications of this sex limitation are discussed in the Limitations section.**

Comment 13: Lines 119-120: You state that allocation and assessment were blinded, but you do not describe the randomization procedure (e.g., block randomization, random number generator) or whether cage effects were controlled. Adding a brief description would strengthen rigor.

Response:

We have added a brief description of the randomization procedure. We also retained the statement that outcome assessments and statistical analyses were performed blinded to group allocation.

Revision:

Lines 128-130

Animals were randomly allocated to experimental groups using a random number generator.

Investigators conducting behavioral scoring, histological assessments, and statistical analyses were blinded to group allocation.

Comment 14: Lines 122-131: Criteria for successful MCAO is "reduction in cerebral blood flow to below 60% of the baseline level, maintained for 60 minutes." You do not state whether animals failing to meet this criterion were excluded and, if so, how many. A CONSORT-style statement on exclusions is important (e.g., number excluded for surgical reasons, death, failure in flow reduction).

Response:

Thank you for this important suggestion. We have added the exclusion numbers for animals excluded because of unsuccessful MCAO model establishment or death during the surgical procedure.

Revision:

Lines 141-142

A reduction in cerebral blood flow to below 60% of the baseline level, maintained for 60 minutes, was considered indicative of successful model establishment. **Across all MCAO experiments, 10 mice were excluded because of unsuccessful model establishment, and 11 mice were excluded because of death during the surgical procedure.**

Comment 15: Lines 132-137: You measure CRH in plasma, which is unconventional and technically challenging; it is important to specify (1) whether this is serum or plasma, (2) validation of the CRH assay in mouse samples (e.g., detection limit, cross-reactivity), and (3) whether samples were run in duplicate/triplicate and intra/inter-assay CV. As CRH is a central mechanistic endpoint, analytical reliability is critical.

Response and revision:

Thank you for this important comment. We agree that the analytical reliability of CRH measurement is critical. We have clarified that hormone levels were measured in serum rather than plasma, and that all ELISA measurements were performed in duplicate. In addition, because circulating CRH levels can be low and variable in serum, we also assessed hypothalamic CRH expression by immunofluorescence staining as a central readout to support the peripheral hormone measurements.

Comment 16: Lines 155-161: You describe collection and sequencing platform, but important details are missing:

Which 16S region (e.g., V3-V4) and primer set were used?

How were reads processed (e.g., DADA2, QIIME2)?

Which reference database and taxonomy classifier?

Were rarefaction depths standardized across samples?

Without these information, reproducibility is limited.

Response:

Thank you for this helpful suggestion. We have expanded the Gut Microbiome Analysis section to include the amplified 16S region and primer set, read-processing pipeline, reference database, taxonomy assignment method, and sequencing-depth normalization. These additions improve the reproducibility of the microbiome analysis.

Revision:

Lines 170-180

Gut microbiota profiling was conducted through 16S rRNA amplicon sequencing on the Illumina NovaSeq platform by Hebei LabMed Technology Co., Ltd. (Hebei, China). The V4 region of the bacterial 16S rRNA gene was amplified using barcoded 515F/806R primers. Paired-end reads were demultiplexed according to barcode and primer sequences, merged using FLASH v1.2.11, processed with Cutadapt to remove residual primer sequences, and quality-filtered using fastp v0.23.1. Chimeric sequences were removed by comparison with the reference database, and effective tags were denoised using the DADA2 module in QIIME2 to generate amplicon sequence variants. Taxonomic annotation was performed in QIIME2 against the SILVA 138.1 database. All samples were normalized to the minimum sequencing depth before alpha- and beta-diversity analyses.

Comment 17: Lines 163-177, 179-188: Methods are fairly detailed, but you do not describe quantification procedures (e.g., how many sections per animal, how many fields per section, selection criteria, and whether thresholding/analysis were done blinded). These details should be stated here, not only in figure legends.

Response:

Thank you for this helpful suggestion. We have added the quantification procedures for immunofluorescence and TUNEL staining, including the number of fields analyzed, ImageJ-based quantification, and blinded analysis.

Revision:

Lines 201-204

For immunofluorescence quantification, three non-overlapping fields per animal were analyzed in predefined regions of interest. Fluorescence intensity was quantified using ImageJ by an investigator blinded to group allocation.

Lines 215-217

For TUNEL quantification, three non-overlapping fields per animal were analyzed in the infarct core. TUNEL-positive cells were expressed as a percentage of DAPI-positive nuclei, and analysis was performed blinded to group allocation.

Comment 18: Lines 190-199: FMT protocol is standard; however you do not mention whether recipient mice received antibiotics prior to FMT, which is common to facilitate colonization. This omission is important because colonization efficiency and interpretation of microbiota changes differ with/without antibiotic preconditioning.

Response:
Response: Thank you for this important comment. We have clarified in the FMT protocol that recipient mice did not receive antibiotic pretreatment before FMT. The intervention was therefore performed in the context of an intact resident microbiota, which should be considered when interpreting colonization efficiency and microbiota changes.

Revision:

Lines 226

Recipient mice did not receive antibiotic pretreatment before FMT

Comment 19: Lines 201-209: You justify AKK dose with a previous study but explicitly state that "The dose-response relationship was not examined in the present study." This should be acknowledged again as a limitation later (dose not optimized, potential under/overdosing).

Response:
We have added this point to the Limitations section, noting that the AKK dose was based on a previous study but was not optimized in the present work, and that potential under- or overdosing cannot be excluded.

Revision:

Limitation

Second, both FMT and AKK treatment were administered before MCAO as preventive interventions. While this design enabled us to explore how microbial modulation influences pre-stroke endocrine status and subsequent outcomes, it does not fully recapitulate the clinical context, where therapeutic interventions typically occur after stroke onset. Therefore, the preventive nature of our design limits its direct translational applicability. **In addition, the AKK dose was selected based on a previous study, but dose-response effects were not examined; therefore, the dose may not represent the optimal therapeutic dose, and potential under- or overdosing cannot be excluded.** Future studies should evaluate post-stroke treatment paradigms to determine the therapeutic potential and optimal intervention

window of microbiota-based strategies.

Comment 20: Lines 227-234: You describe body weight and behavioral deficits; however, absolute body weight values, infarct size, and baseline performance are not reported in text. Consider including key numbers (e.g., mean weight {plus minus} SEM, Longa scores) to give the reader a sense of effect size.

Response:

Thank you for this helpful suggestion. We have added key numerical values to the Results section, including absolute body weight and Longa scores at each post-stroke time point, to provide a clearer sense of effect size.

Revision:

3.1 High-Fat Diet Worsens Post-Ischemic Neurological Outcomes and Is Associated with HPA Axis Dysfunction

To determine whether dietary regimens influenced neurological outcomes after MCAO, we first assessed functional deficits in mice fed distinct diets. As shown in Figure 1a, HFD-fed mice exhibited a significant increase in body weight compared to ND controls **(ND: 28.01 ± 0.47 g; HFD: 33.67 ± 0.85 g)** after 8 weeks of dietary intervention. Neurological function was systematically assessed at days 1, 3, and 7 post-MCAO. The rotarod test revealed significantly impaired motor coordination and endurance in HFD mice compared to ND mice (Figure 1b). **Furthermore, Longa scores were higher in HFD mice than in ND mice at each post-stroke time point (Day 1: ND, 2.20 ± 0.29; HFD, 2.70 ± 0.33; Day 3: ND, 1.70 ± 0.15; HFD, 2.30 ± 0.21; Day 7: ND, 1.00 ± 0.15; HFD, 1.80 ± 0.25), indicating more severe neurological impairment in the HFD group.** (Figure 1c). The open field test showed reduced locomotor activities in HFD mice, evidenced by shorter total movement distances (Figure 1d, e). These results indicate that HFD exacerbates post-ischemic neurological impairment compared to ND.

Comment 21: Lines 237-239: "HFD mice exhibited significantly lower levels of corticosterone, ACTH, and CRH levels at all time points compared to normal diet controls" is strong; indicate whether baseline pre-MCAO hormone levels already differed (they did by design, but emphasize this) and whether there is any time × diet interaction. A simple "at all time points" hides potential differences in dynamic response vs baseline shift.

Response:

Thank you for this helpful comment. The 0 h time point in the hormone curves represents the pre-MCAO baseline level. Thus, HFD mice already showed lower corticosterone, ACTH, and CRH levels before ischemic injury, and this baseline difference was maintained across the

post-MCAO time points. This baseline alteration was the main reason why, in the subsequent FMT and AKK intervention experiments, we focused primarily on pre-MCAO baseline hormone levels to assess whether microbiota intervention could restore the HPA-axis endocrine state before stroke. We agree that the phrase "at all time points" should be interpreted as reflecting both a baseline shift and post-MCAO group differences, rather than a separate conclusion about time × diet interaction.

Comment 22: Lines 241-243: Correlation R^2 values (0.35 and 0.22) show modest relationships; mention that correlations, while significant, explain only a portion of variance. Avoid overinterpreting as strong predictive relationships.

Response:
Thank you for this helpful comment. We have revised the Results section to clarify that, although the correlations between HPA-axis hormone levels and Longa scores were statistically significant, the R^2 values indicate only modest explanatory power. We have therefore avoided interpreting these associations as strong predictive relationships.

Revision:

Lines 276-278
Although these correlations were statistically significant, the R^2 values indicate that CRH and corticosterone levels explained only a modest proportion of the variance in neurological deficit scores.

Comment 23: Lines 261-264: You conclude that reduced hypothalamic CRH expression "suggests" impaired HPA output. This is plausible but you do not distinguish between CRH neuron number loss vs reduced expression per neuron. Clarify in the text what exactly was quantified (cell counts vs mean fluorescence intensity) and whether region boundaries were precisely defined.

Response:
Thank you for this helpful comment. We have clarified that hypothalamic CRH was quantified as mean fluorescence intensity within a predefined hypothalamic region of interest, rather than by counting CRH-positive neurons. We have also softened the interpretation to state that reduced CRH immunoreactivity suggests decreased regional CRH expression, while acknowledging that this analysis cannot distinguish reduced expression per neuron from changes in CRH-positive cell number.

Revision:

Lines 284-286
Immunofluorescence staining for CRH in the hypothalamus revealed markedly reduced CRH expression in HFD-fed mice compared to those fed a normal diet. This observation suggests

that HFD may impair the functional output of the HPA axis by downregulating hypothalamic CRH expression, potentially affecting stress responsiveness and hormonal regulation, **but this analysis does not distinguish reduced expression per neuron from changes in CRH-positive cell number.**

Comment 24: Lines 269-275: Microglial and TUNEL data are described qualitatively but again without numerical values or effect sizes. Adding the magnitude of changes (% increase in Iba-1 intensity, fold change in TUNEL+ cells) would help readers assess biological relevance.

Response:

Thank you for this helpful suggestion. We have added numerical values and fold-change estimates for Iba-1 fluorescence intensity and TUNEL-positive cells to better indicate the magnitude and biological relevance of these effects.

Revision:

Lines 291-292

The HFD group exhibited significantly higher Iba-1 fluorescence intensity compared to the ND group **(1.45 ± 0.12 vs. 1.00 ± 0.06)**

Lines 296-297

The number of TUNEL-positive cells was markedly elevated in HFD-fed mice relative to ND controls **(0.67 ± 0.07 vs. 0.47 ± 0.03)**

Comment 25: Lines 291-297: You show that HFD reduces alpha diversity and alters beta diversity. It would be useful to explicitly state which diversity indices show significant differences (Chao1 and observed features are mentioned, but p-values are not given in text).

Response:

Thank you for this helpful suggestion. We have supplemented the corresponding p-values for the alpha-diversity indices in the Results section. We have also updated Figure 3 to include additional alpha-diversity measures, including Chao1, observed features, Shannon, and Simpson indices, to more clearly show the effects of HFD on gut microbial diversity.

Revision:

We revised Figure 3 and added the requested alpha-diversity plots, including Chao1, observed features, Shannon, and Simpson indices.

To investigate the impact of a high-fat diet on the gut microbiota of mice, fecal samples were collected one day prior to MCAO surgery for 16S rRNA amplicon sequencing analysis. The

Venn diagram revealed a total of 382 ASVs shared between the HFD and ND groups, with 1,174 unique ASVs identified in the ND group and 840 unique ASVs in the HFD group (Figure 3b). Alpha diversity analysis indicated that microbial richness was notably reduced in HFD-fed mice compared to those on a normal diet, **as reflected by lower Chao1 (p=0.0004), observed features (p=0.004), Shannon index (p=0.016), and Simpson index (p= 0.008) (Figure 3a).** Beta diversity analysis further demonstrated significant differences in microbial community composition between the two groups (Figure 3d). These results suggest that 8 weeks of HFD feeding markedly reduced both the richness and diversity of the gut microbiota in mice. Multilevel taxonomic analysis of microbial composition revealed that, at the phylum level, the relative abundances of Firmicutes, Actinobacteria, and Desulfobacterota were significantly increased in the HFD group, whereas the abundances of Verrucomicrobiota and Lactobacillota were significantly decreased compared to the ND group (Figure 3d,e). Further analysis at the genus level showed a significant reduction in the abundance of Akkermansia in the HFD group relative to the ND group (Figure 3d,f). Collectively, these findings indicate that the gut microbial composition in HFD-fed mice differs markedly from that in mice maintained on a normal diet.

Comment 26: Lines 299-301: The phylum-level shifts (increased Firmicutes, Actinobacteria, Desulfobacterota; decreased Verrucomicrobiota, Lactobacillota) are described but without mentioning statistical test or correction; clarify that these differences are statistically significant and indicate whether you controlled for multiple testing

Response:

Response: Thank you for this helpful suggestion. We have revised Figure 3 to include quantitative comparisons of representative bacterial phyla and genera, rather than only showing stacked bar plots.

Revision:

Figure3

We revised Figure 3 by adding quantitative analysis plots for representative bacterial phyla and genera.

Comment 27: Lines 302-304: Akkermansia reduction is central to the intervention concept; consider quantifying its relative abundance (e.g., median %, IQR) to emphasize the magnitude of depletion.

Response:

We have revised Figure 3 to include quantitative comparisons of representative bacterial phyla and genera, including Akkermansia, rather than only showing stacked bar plots.

Revision:

Figure3

We revised Figure 3 by adding quantitative analysis plots for representative bacterial phyla and genera, including *Akkermansia*.

Comment 28: Lines 325-325: HFD mice receiving FMT or AKK had reduced body weight compared with HFDPBS. This is important because it introduces a potential confounder: improved outcomes may partially reflect weight loss/metabolic improvement rather than solely microbiota-HPA effects. You should acknowledge this in the Results (not only Discussion) and ideally adjust or at least discuss in terms of metabolic parameters.

Response:

Thank you for this important comment. We agree that the reduced body weight after FMT or AKK administration may represent a potential confounder. We have added a statement in the Results section to acknowledge that the improved hormone levels and stroke outcomes should be interpreted in the context of concomitant metabolic changes. We have also expanded the Limitation section to note that future studies with weight-matched controls and additional metabolic parameters are needed to disentangle microbiota-HPA-axis effects from general metabolic improvement.

Revision:

Lines 335-337

Because both FMT and AKK administration reduced body weight in HFD mice, the subsequent improvements in hormone levels and stroke outcomes should be interpreted in the context of concomitant metabolic changes, rather than being attributed solely to microbiota-HPA-axis modulation.

Limitation

Besides, the reduction in body weight after FMT or AKK administration represents a potential confounding factor, as improved neurological outcomes may partly reflect weight loss or broader metabolic improvement.

Comment 29: Lines 327-333: You state that ACTH and corticosterone are significantly elevated, whereas CRH increases but does not reach significance in HFDFMT. This suggests that CRH might not be the primary driver of peripheral hormone changes, or that power is limited; you should note this nuance explicitly and avoid presenting CRH restoration as equally robust in all groups later.

Response:

We agree that the CRH result in the HFD^{FMT} group should be interpreted more cautiously. We have revised the Results section to explicitly state that FMT significantly increased ACTH and corticosterone levels, whereas serum CRH showed only a non-significant increasing trend. We have also revised later descriptions to avoid presenting CRH restoration as equally robust across all intervention groups, and now describe the FMT effect as a partial restoration of HPA-axis hormone profiles.

Revision:

Lines 345-346
Thus, FMT appeared to partially restore the HPA-axis hormone profile, with more robust effects on ACTH and corticosterone than on serum CRH.

Comment 30: Lines 335-342: Longa and rotarod improvements are described, with AKK showing earlier, stronger effects than FMT. It would help to state effect sizes (e.g., mean difference in Longa score vs HFDPBS on day 7) and mention whether there were interactions between time and treatment (since improvements emerge over days).

Response:
Thank you for this helpful suggestion. We have revised the Results section to include representative effect-size information for the behavioral outcomes, focusing on the Day 7 differences in Longa score and rotarod latency relative to HFDPBS controls.

Revision:

Behavioral performance was evaluated at 1, 3, and 7 days after reperfusion. Longa neurological scores showed that both HFDFMT and HFDAKK groups had significantly lower scores than HFDPBS controls, reflecting improved neurological recovery (Figure 4f). **This difference was evident on day 7, when Longa scores were reduced by 0.70 points in the HFDFMT group and 0.90 points in the HFDAKK group compared with HFDPBS mice.** In the rotarod test, latency to fall was significantly prolonged in the HFDAKK group at all examined time points, indicating consistently enhanced motor coordination. In contrast, the HFDFMT group exhibited a trend toward improvement during the early phase, which became statistically significant on day 7. **At this time point, rotarod latency was increased by 63.3 s in HFDFMT mice and 84.5 s in HFDAKK mice compared with HFDPBS controls (Figure 4g)**. No significant differences were found between the HFDAKK and HFDFMT groups in either neurological scores or rotarod performance at any time point.

Open-field testing further supported these behavioral results. Representative trajectory plots and heatmaps revealed greater spontaneous locomotor activity in both intervention groups compared with HFDPBS controls. Quantitative analysis showed that total movement distance was significantly greater in the HFDAKK group at all time points after MCAO, whereas the

HFDFMT group showed increased locomotion with a statistically significant difference on day 7 (Figure 4h–i). **On day 7, total movement distance was approximately doubled in the HFDFMT group and increased by more than two-fold in the HFDAKK group relative to HFDPBS controls.**

Comment 31: Lines 349-352: You state "Although elevated HPA-axis hormones accompanied functional improvement, the present data do not establish a causal relationship." This is an important caveat and might be moved forward in the paragraph to prevent readers from inferring causality earlier.

Response:
Thank you for this helpful suggestion. We agree that this caveat should be stated earlier to avoid overinterpretation. We have moved this statement forward in the paragraph and revised the wording to clarify from the outset that the observed increase in HPA-axis hormones accompanied, but does not prove causally mediate, the improved behavioral outcomes.

Revision:

Lines 349-353

Although elevated HPA-axis hormones accompanied functional improvement, the present data do not establish a causal relationship between hormone restoration and behavioral recovery. Therefore, the improved outcomes after FMT or AKK administration should be interpreted as being associated with, rather than directly caused by, changes in HPA-axis activity.

Lines 373-375

Together, these findings suggest that FMT and AKK administration were associated with improved post-stroke functional recovery and partial restoration of HPA-axis hormone profiles in HFD mice, with AKK showing relatively earlier and more consistent behavioral effects.

Comment 32: Lines 371-376: You refer to "Figure S1a-c" for CRH restoration after FMT and AKK, but these are supplementary figures; given that CRH is a central mechanistic link, consider moving these data into main figures or at least referencing them more strongly.

Response:
Thank you for this helpful suggestion. We agree that hypothalamic CRH expression is an important mechanistic readout in this study. We have revised the main text to refer more explicitly to these data and to emphasize that FMT and AKK administration increased hypothalamic CRH immunoreactivity, supporting partial restoration of central HPA-axis

activity. We retained these images in the Supplementary Figure to avoid overloading the main figures, but strengthened their description in the Results section.

Revision:

Lines 382-384

First, consistent with the peripheral hormone results, hypothalamic CRH immunofluorescence further showed increased CRH immunoreactivity after FMT and AKK administration, supporting partial restoration of central HPA-axis activity in HFD mice.

Comment 33: Lines 378-383: Iba-1 and TUNEL changes are presented, but the phrase "more notable reduction" and "a decreasing trend (p = 0.0574)" risks overemphasizing near-significant results. It is better to state clearly that TUNEL reduction in HFDFMT did not reach significance and treat it as hypothesis-generating rather than quasi-positive.

Response:

We agree that the previous wording may have overemphasized the near-significant TUNEL result in the HFD^FMT group. We have revised the Results section to clearly state that TUNEL reduction after FMT did not reach statistical significance.

Revision:

Lines 395-398

whereas the reduction in the HFDFMT group did not reach statistical significance (p=0.0574). These findings suggest that AKK administration may mitigate post-ischemic neuronal apoptosis, whereas the potential anti-apoptotic effect of FMT requires further confirmation (Figure S2a–c).

Comment 34: Lines 399-402: Alpha diversity is reduced in intervention groups compared with HFDPBS. This is a paradoxical finding given that interventions are beneficial; you should highlight this tension more clearly and avoid implying that diversity restoration is the mechanism of benefit.

Response:

Thank you for this important comment. We agree that the reduced alpha diversity after FMT or AKK administration should not be interpreted as diversity restoration. We have revised the Discussion to explicitly highlight this apparent tension and to clarify that the beneficial effects of the interventions are more likely related to specific compositional or functional microbial changes, rather than an overall increase in alpha diversity.

Revision:

Discussion

The observed associations, although statistically significant, do not confirm direct endocrine causality. **Notably, alpha diversity was reduced after FMT and AKK administration compared with HFDPBS mice, despite the beneficial effects of these interventions on hormone levels and post-stroke outcomes. Therefore, these findings should not be interpreted as evidence that microbiota intervention improved recovery by restoring overall microbial diversity. Instead, the protective effects may be more closely related to specific compositional or functional changes, such as Akkermansia-associated alterations and modulation of gut-brain neuroendocrine signaling.** Prior evidence supports the notion that microbial components and metabolites can influence HPA axis regulation

Comment 35: Lines 404-406: The phylum-level differences in HFDAKK (higher Proteobacteria) might raise concerns about potential pro-inflammatory consequences; this deserves explicit mention and cautious interpretation.

Response:

We have revised the Discussion to explicitly acknowledge the increased relative abundance of Proteobacteria in the HFD^AKK group and to interpret this finding cautiously. We now note that, although AKK administration was associated with improved behavioral and neuroendocrine outcomes, its effects on microbial composition are complex and should not be interpreted as uniformly beneficial.

Revision:

Discussion

In addition, the increased relative abundance of Proteobacteria in the HFDAKK group should be interpreted cautiously, as expansion of this phylum is often associated with inflammatory or dysbiotic states. Although AKK administration improved behavioral and neuroendocrine outcomes in the present study, this compositional change indicates that its microbiota-level effects are complex and not uniformly beneficial.

Comment 36: Lines 420-429: The summary of findings is accurate but somewhat overstated in mechanistic certainty ("interventions...partially restored CRH expression..., elevated systemic glucocorticoid levels, and improved...outcomes"). You should reiterate that causality is not demonstrated and that HPA changes may be one of several mediating pathways.

Response:

We agree that the mechanistic interpretation should be more cautious. We have revised the Discussion to clarify that FMT and AKK administration were associated with, rather than proven to cause, partial restoration of HPA-axis activity and improved outcomes.

Revision:

Lines 424-428

More importantly, **our findings show that FMT and AKK administration were associated with partial restoration of hypothalamic CRH immunoreactivity, increased circulating HPA-axis hormone levels, and improved post-stroke functional outcomes in HFD mice. However, these data do not establish a causal relationship between HPA-axis changes and functional recovery.**

Comment 37: Lines 435-446: You nicely acknowledge that association does not prove endocrine causality. However, later sentences (e.g., lines 441-443) risk reintroducing a causal tone ("may serve...as modulators of central neuroendocrine circuits") without clearly framing it as speculative. Consider adding explicit wording like "We speculate that..." or "It is plausible but remains unproven that...".

Response:

We have revised the wording to explicitly frame the proposed modulation of central neuroendocrine circuits as speculative, using "We speculate that" to avoid implying direct mechanistic proof.

Revision:

Lines 448

We speculate that specific microbial taxa, such as Akkermansia, may serve not only as anti-inflammatory agents but also as modulators of central neuroendocrine circuits.

Comment 38: Lines 448-459: The discussion of endocrine-independent pathways is strong, but you may want to explicitly highlight that, given modest corticosterone changes and strong anti-inflammatory effects, non-HPA mechanisms may account for a substantial portion of benefit.

Response:

We have revised the Discussion to more explicitly acknowledge that non-HPA mechanisms may contribute substantially to the beneficial effects of FMT and AKK administration, particularly given the modest changes in corticosterone and the stronger anti-inflammatory and anti-apoptotic effects observed in the study.

Revision:

Lines 461-463

Given the modest changes in circulating corticosterone and the pronounced reduction in inflammatory and apoptotic markers, non-HPA mechanisms may account for a substantial portion of the observed benefit after FMT or AKK administration.

Comment 39: Lines 462-469: You discuss reduced alpha diversity in interventions but do not critically reflect on whether this may carry risks or reflect dominance of a few taxa (including Proteobacteria). Adding a short caveat would demonstrate balanced interpretation.

Response:

We have added a short caveat to the Discussion noting that reduced alpha diversity after intervention may reflect dominance of selected taxa, including Proteobacteria, and that the potential consequences of this compositional shift should be interpreted cautiously.

Revision:

Lines 474-475

While alpha diversity was reduced in the intervention groups-perhaps due to the selective colonization of dominant taxa-β diversity analyses indicated a clear shift in overall composition. **This reduced diversity may reflect dominance of selected taxa, including Proteobacteria, and its potential long-term consequences require cautious interpretation.**

Comment 40: Lines 471-472: Suggesting gnotobiotic and defined consortia approaches is appropriate; you might also suggest metabolomics and targeted SCFA or bile acid analysis as next steps.

Response:

We have revised the Discussion to include metabolomics and targeted SCFA or bile acid analyses as important future directions, in addition to gnotobiotic models and defined microbial consortia.

Revision:

Lines 482-484

Future studies using gnotobiotic models, defined microbial consortia, metabolomics, and targeted SCFA or bile acid analyses will be needed to identify the specific microbial metabolites and pathways involved.

Comment 41: Lines 474-482: The idea that patients with suppressed HPA axis may benefit from microbiota-based approaches is interesting but highly speculative at this stage. You should explicitly say that clinical HPA suppression in obese/MetS stroke patients has not yet been systematically linked to microbiota signatures in the way your model suggests, and that translation will require substantial validation.

Response:

We have revised the Discussion to clarify that this clinical implication remains speculative

and that substantial clinical validation will be needed before microbiota-based approaches can be translated to obese or metabolic syndrome stroke patients.

Revision:

Lines 495-498

However, this clinical implication remains speculative. HPA-axis suppression in obese or metabolic syndrome stroke patients has not yet been systematically linked to specific gut microbiota signatures, and substantial clinical validation will be needed before translation.

Comment 42: Lines 478-481: The sentence beginning "Moreover, Emerging concepts in stroke medicine..." has capitalization and stylistic issues ("Emerging" should not be capitalized; "Moreover," is unnecessary).

Response:

Thank you for pointing this out. We have corrected the capitalization issue and removed the unnecessary “Moreover,” to improve the style and readability of the sentence.

Revision:

Lines 498

remove “Moreover”

Comment 43: Lines 486-495: The sex limitation is well discussed, but you might also highlight that Akkermansia-HPA interactions could be sex-specific, making male-only data particularly hard to generalize.

Response:

We have revised the Limitations section to further emphasize that Akkermansia–HPA-axis interactions may be sex-specific, which limits the generalizability of the present male-only findings.

Revision:

Lines 509-511

Moreover, Akkermansia-HPA axis interactions may be sex-specific, which further limits the generalizability of the present male-only findings.

Comment 44: Lines 497-502: You acknowledge the preventive design; this is appropriate. You might also mention that the 7-day pre-treatment window is relatively short and does not model long-standing microbiome modulation.

Response:

We have revised the Limitations section to note that the 7-day pretreatment window was relatively short and may not fully represent long-standing microbiome modulation.**Revision:**

Lines 519-522

Therefore, the preventive nature of our design, together with the relatively short 7-day pretreatment window, limits its direct translational applicability and may not fully model long-standing microbiome modulation.

Comment 45: Lines 504-507: You mention that mechanistic relationships remain incompletely understood; this could be strengthened by explicitly acknowledging the absence of HPA blockade experiments (e.g., glucocorticoid receptor antagonists, adrenalectomy) and lack of direct CRH neuron manipulation.

Response:
We have revised the Limitations section to clarify that the mechanistic relationship between gut microbial alterations, CRH regulation, and corticosterone secretion remains incompletely understood and requires further confirmation using direct approaches.

Revision:

Lines 533-535
In addition, the mechanistic relationship between gut microbial alterations, CRH regulation, and corticosterone secretion remains incompletely understood a**nd requires further confirmation using direct approaches, such as HPA-axis blockade or targeted modulation of CRH signaling.**

Comment 46: Missing limitation:

No metabolic phenotyping beyond body weight (e.g., glucose, insulin, lipids) is provided, limiting interpretation of how "metabolic stress" was modulated by interventions. Adding a note about missing metabolic endpoints would strengthen transparency.

Antibiotics not used before FMT (if true) could be explicitly acknowledged as a factor that may limit colonization efficiency and generalizability to standard FMT protocols.

Response:
Thank you for these helpful suggestions. We have revised the Limitations section to acknowledge that metabolic phenotyping was limited to body weight and did not include glucose, insulin, lipid profiles, or other metabolic endpoints. We have also explicitly noted that recipient mice did not receive antibiotic pretreatment before FMT, which may have limited donor microbiota engraftment and may affect generalizability to standard FMT protocols.

Revision:

Limitation

In addition, the mechanistic relationship between gut microbial alterations, CRH regulation, and corticosterone secretion remains incompletely understood and requires further confirmation using direct approaches, such as HPA-axis blockade or targeted modulation of CRH signaling. Metabolic phenotyping was also limited to body weight, without glucose, insulin, lipid, or other metabolic endpoints, limiting interpretation of metabolic changes after intervention. Moreover, recipient mice did not receive antibiotic pretreatment before FMT, which may have affected donor microbiota engraftment. Further research integrating metabolic assessment and causal manipulation approaches is required to clarify whether microbiota-induced hormonal changes directly mediate neuroprotection after stroke.

Reviewer 2

Public repository details (Required)：Gut microbiome data need to be deposited.

Response:

Thank you for this requirement. We have added a Data Availability statement and will deposit the 16S rRNA sequencing data in a public repository before publication. The accession number will be provided once the submission to the repository is completed.

Comment 1: The current conclusions, particularly those regarding therapeutic implications for stroke, are overconcluded given that the study relies exclusively on male data. Biological sex is a critical variable in stroke pathophysiology and treatment response; therefore, the results cannot be assumed to apply to female cohorts without direct investigation. The study design was only based on male mice therefore therapeutic implications for stroke patients cannot legitimately apply to female patients on the basis of male-only data. The abstract , discussion and throughout the draft need a revision to explicitly limit all conclusions to male animals.

Response:

Thank you for this important comment. We fully agree that the use of male mice only limits the generalizability of the present findings. We have revised the Abstract, Discussion, Conclusion, and relevant sections throughout the manuscript to explicitly state that our conclusions are based on male mice and should not be assumed to apply to females without direct investigation. We have also expanded the Discussion to address this limitation in more detail, emphasizing that biological sex is a critical variable in stroke pathophysiology, HPA-axis regulation, microbiota composition, and treatment response.

Comment 2: The findings in this study were mainly correlational not a direct causation. I suggest the authors to revise the title, abstract, and discussion to use more conservative, association-based language (e.g., "is associated with" or "correlates with") rather than strongly address a direct cause, which could lead to scientifically misleading.

Response:

Thank you for this important comment. We agree that the present findings are mainly correlational and do not establish direct causality. This issue was also raised by Reviewer 1, and we have revised the title, Abstract, Discussion, Conclusion, and relevant sections throughout the manuscript to use more conservative, association-based language.

Revision:

Lines 349-352

Although elevated HPA-axis hormones accompanied functional improvement, the present data do not establish a causal relationship between hormone restoration and behavioral recovery. Therefore, the improved outcomes after FMT or AKK administration should be interpreted as being associated with, rather than directly caused by, changes in HPA-axis activity.

Lines 373-376

Together, these findings suggest that FMT and AKK administration were associated with improved post-stroke functional recovery and partial restoration of HPA-axis hormone profiles in HFD mice, with AKK showing relatively earlier and more consistent behavioral effects.

Lines 424-428

More importantly, **our findings show that FMT and AKK administration were associated with partial restoration of hypothalamic CRH immunoreactivity, increased circulating HPA-axis hormone levels, and improved post-stroke functional outcomes in HFD mice. However, these data do not establish a causal relationship between HPA-axis changes and functional recovery.**

Lines 448

We speculate that specific microbial taxa, such as Akkermansia, may serve not only as anti-inflammatory agents but also as modulators of central neuroendocrine circuits.

Comment 3: The data shown in the Result section were based only on behavioural scores (Longa, rotarod, open field) and inflammatory markers without establishing whether these differences are accompanied by proportional differences in infarct size. If HFD mice have larger infarcts, some or all of the behavioural deficit could be explained by greater tissue loss rather than impaired recovery. Could the authors add infarct volume as percentage of ipsilateral hemisphere to strengthen this observation?

Response:

Thank you for this helpful suggestion. We agree that infarct volume would provide an

important anatomical measure to further support the behavioral findings. However, infarct volume was not quantified in the present study, and the available tissue sections were primarily used for immunofluorescence and TUNEL analyses. We have therefore added this point to the Limitations section, noting that the absence of infarct volume measurement limits our ability to determine whether behavioral deficits were partly driven by differences in tissue loss.

Revision:

Lines 535-536

Infarct volume was not directly quantified, limiting anatomical interpretation of the behavioral outcomes.

Comment 4: In this study, the authors proposed that gut dysbiosis suppresses CRH through gut-barrier dysfunction and TLR4/NF-κB signalling, or via microbial metabolites, yet none of these intermediate steps were measured. Please elaborate on this matter or revise the draft to conclude only evidence supported by the data.

Response:
Thank you for this comment. We would like to clarify that gut-barrier dysfunction, TLR4/NF-κB signaling, and microbial metabolites were discussed in the Discussion based on prior evidence, rather than presented as mechanisms directly demonstrated in the present study. For example, the paragraph begins with “Prior evidence supports the notion that microbial components and metabolites can influence HPA axis regulation.” To avoid any misunderstanding, we have further revised the wording to make clear that these pathways are literature-based possible mechanisms and were not directly assessed in our model.

Revision:

Lines 441-443

Prior evidence supports the notion that microbial components and metabolites can influence HPA-axis regulation, although these intermediate pathways were not directly assessed in the present study

Comment 5: Iba-1 fluorescence intensity is useful as a proxy for microglial morphological activation. It could not distinguish pro- from anti-inflammatory states (M1/M2 polarisation), or capture infiltrating monocytes or other immune effectors. Therefore, the mentioned exacerbated neuroinflammation was derived based on a single histological marker without systemic inflamatory data. Could the authors further explain or clarify this conclusion?

Response:
Thank you for this important comment. We agree that Iba-1 fluorescence intensity alone

cannot fully characterize neuroinflammation or distinguish microglial polarization states and other immune effectors. We have therefore revised the wording throughout the manuscript to describe these findings more specifically as changes in Iba-1-positive microglial activation, rather than as a comprehensive measure of neuroinflammation.

Comment 6: The main key from this study was that microbiota intervention could restore HPA axis activity and that this hormonal restoration could improved stroke outcomes. This is a bit contradicted by the FMT data. Figure 4e shows that CRH levels in the HFD-FMT group do not significantly differ from HFD-PBS controls ($p > 0.05$; described as a "trend"). Yet the same group shows significantly improved Longa scores, rotarod performance, and open-field distance (Figs. 4f-i) and reduced Iba-1 staining (Fig. 5). If CRH restoration is the mechanism, how did a group without significant CRH elevation achieve equivalent behavioural benefit? Perhaps, the authors can discuss this discrepancy.

Response:
Thank you for this important comment. We agree that the FMT results indicate that behavioral improvement cannot be explained solely by significant serum CRH restoration. We have revised the Discussion to explicitly acknowledge this discrepancy and to clarify that non-HPA mechanisms may also contribute substantially to the observed benefits. Specifically, we now state that, given the modest changes in circulating corticosterone and the pronounced reduction in inflammatory and apoptotic markers, non-HPA mechanisms may account for a substantial portion of the benefit after FMT or AKK administration.

Revision:

Lines 461-463

Given the modest changes in circulating corticosterone and the pronounced reduction in inflammatory and apoptotic markers, non-HPA mechanisms may account for a substantial portion of the observed benefit after FMT or AKK administration.

Comment 7: A single dose of 2×10^9 CFU/200 μL was used throughout, justified by reference to one prior study with a different disease model. Please elaborate more or add rationale why this single dose was selected.

Response:
Thank you for this helpful comment. We have added a rationale and limitation regarding the AKK dose in the Discussion. We now clarify that the dose was selected based on a previous study, but dose-response effects were not examined in the present work. Therefore, this dose may not represent the optimal therapeutic dose, and potential under- or overdosing cannot be excluded.

Revision:

Lines 526-529

The AKK dose was selected based on a previous study, but dose-response effects were not examined; therefore, the dose may not represent the optimal therapeutic dose, and potential under- or overdosing cannot be excluded.

Comment 8: Methods section (lines 163-178) only showed antibody concentrations and excitation/emission wavelengths but omits instrument settings critical for reproducibility of fluorescence intensity comparisons. Please add more details.

Response:

Thank you for this helpful suggestion. We have expanded the Methods section to include additional imaging parameters relevant to fluorescence intensity comparison. Specifically, we clarified that images were acquired using a 40× objective and that exposure time, gain, light intensity, and image resolution were kept constant across groups within the same staining batch.

Revision:

Lines 199-201

Fluorescence images were acquired using a 40× objective. For each marker, exposure time, gain, light intensity, and image resolution were kept constant across all groups within the same staining batch.

Comment 9: Lines 129-131 state the criterion but lines 102-103 present group sizes without accounting for surgical failures. I suggest the authors adding a statement in Methods or Results reporting the number of animals per group that met the CBF success criterion, the number excluded, and the total enrolled. Report whether CBF monitoring was continuous or sampled.

Response:

Response: We have revised the Methods section to clarify animal exclusions during MCAO experiments. Specifically, we now state that, across all MCAO experiments, 10 mice were excluded because of unsuccessful model establishment and 11 mice were excluded because of death during the surgical procedure.

Revision:

Lines 141-142

Across all MCAO experiments, 10 mice were excluded because of unsuccessful model establishment, and 11 mice were excluded because of death during the surgical procedure.

Comment 10: Need bioinformatics analysis details (Line 159-161)

Response:

Response: We have supplemented the bioinformatics analysis details in the corresponding Methods section, including read processing, quality filtering, chimera removal, ASV generation, taxonomic annotation, reference database, and sequencing-depth normalization before diversity analyses.

Revision:

Lines 169-182

Fecal sample collection was performed by elevating the tail and applying gentle abdominal pressure to stimulate defecation, freshly expelled feces were collected using sterile forceps and immediately transferred into sterile cryovials, which were subsequently stored at -80°C. Each biological sample contained 2-3 fecal pellets. Gut microbiota profiling was conducted through 16S rRNA amplicon sequencing on the Illumina NovaSeq platform by Hebei LabMed Technology Co., Ltd. (Hebei, China). The V4 region of the bacterial 16S rRNA gene was amplified using barcoded 515F/806R primers. Paired-end reads were demultiplexed according to barcode and primer sequences, merged using FLASH v1.2.11, processed with Cutadapt to remove residual primer sequences, and quality-filtered using fastp v0.23.1. Chimeric sequences were removed by comparison with the reference database, and effective tags were denoised using the DADA2 module in QIIME2 to generate amplicon sequence variants. Taxonomic annotation was performed in QIIME2 against the SILVA 138.1 database. All samples were normalized to the minimum sequencing depth before alpha- and beta-diversity analyses.

Comment 11: For corticosterone assay, please add a time of day for blood collection of each group.

Response:

Thank you for this helpful suggestion. We have clarified that MCAO procedures were performed between 11:00 and 15:00 and that blood samples were collected at predefined time points using the same schedule across groups.

Revision:

Lines 145-147

MCAO procedures were performed between 11:00 and 15:00, and blood samples were collected at predefined time points using the same schedule across groups.

Conclusion

We sincerely thank the reviewers for their constructive and insightful comments. In the revised manuscript, we have substantially improved the clarity and rigor of the study by

limiting conclusions to male mice, using more conservative association-based language, adding methodological details, reporting additional quantitative results, and expanding the discussion of key limitations. We believe these revisions have strengthened the manuscript and improved the transparency and interpretation of our findings.

Re: Spectrum03549-25R1 (**Gut Dysbiosis Impairs HPA Axis Function and Exacerbates Post-Stroke Inflammation in High-Fat Diet-Fed Male Mice**)

Dear Dr. Yunpeng Liu:

Thank you for your revised submission. All previous reviewer comments have been satisfactorily addressed.

Before we can move forward with final processing, please note a required step regarding data policy compliance: ASM policy requires data to be publicly available. If your data is available only upon request, you must include a brief explanation in the comment box under the "ASM Data Policy compliance" question within the Author Certification section in the eJP submission system. These explanations are reviewed on a case-by-case basis.

Once this step is complete, we will proceed with the final decision.

Please return the manuscript within 14 days; if you cannot complete the modification within this time period, please contact me. If you do not wish to modify the manuscript and prefer to submit it to another journal, notify me immediately so that the manuscript may be formally withdrawn from consideration by Spectrum.

Revision Guidelines
To submit your modified manuscript, log into the submission site at https://spectrum.msubmit.net/cgi-bin/main.plex. Go to Author Tasks and click the appropriate manuscript title to begin. The information you entered when you first submitted the paper will be displayed; update this as necessary. Note the following requirements:

• Upload point-by-point responses to the issues raised by the reviewers in a file named "Response to Reviewers," NOT in your cover letter.
• Upload a compare copy of the manuscript (without figures) as a "Marked-Up Manuscript" file.
• Upload a clean .DOC/.DOCX version of the revised manuscript and remove the previous version.
• Each figure must be uploaded as a separate, editable, high-resolution file (TIFF or EPS preferred), and any multipanel figures must be assembled into one file.
• Any supplemental material intended for posting by ASM should be uploaded with their legends separate from the main manuscript. You can combine all supplemental material into one file (preferred) or split it into a maximum of 10 files with all associated legends included.

For complete guidelines on revision requirements, see our Submission and Review Process webpage. Submission of a paper that does not conform to guidelines may delay acceptance of your manuscript.

Data availability: ASM policy requires that data be available to the public upon online posting of the article, so please verify all links to sequence records, if present, and make sure that each number retrieves the full record of the data. If a new accession number is not linked or a link is broken, provide Spectrum production staff with the correct URL for the record. If the accession numbers for new data are not publicly accessible before the expected online posting of the article, publication may be delayed; please contact production staff (Spectrum@asmusa.org) immediately with the expected release date.

Publication Fees: For information on publication fees and which article types are subject to charges, visit our website. If your manuscript is accepted for publication and any fees apply, you will be contacted separately about payment during the production process; please follow the instructions in that e-mail. Arrangements for payment must be made before your article is published.

ASM Membership: Corresponding authors may join or renew ASM membership to obtain discounts on publication fees. Need to upgrade your membership level? Please contact Customer Service at Service@asmusa.org.

The ASM Journals program strives for constant improvement in our submission and publication process. Please tell us how we can improve your experience by taking this quick Author Survey.

Thank you for submitting your paper to Spectrum.

Sincerely,
Wanilada Rungrassamee
Editor
Microbiology Spectrum

Reviewer #2 (Public repository details (Required)):

Data needs to be publicly available.

Reviewer #2 (Comments for the Author):

I have no further comments. However, the authors need to deposit their data to public database.

Response to Reviewers

Manuscript ID: Spectrum03549-25R1

Title: Gut Dysbiosis Impairs HPA Axis Function and Exacerbates Post-Stroke Inflammation in High-Fat Diet-Fed Male Mice

We sincerely thank the reviewers for their careful evaluation of our manuscript and for the constructive comments. We have revised the manuscript substantially to improve clarity, methodological transparency, and interpretive balance. In particular, we have softened causal and translational claims throughout the manuscript, clarified that the findings are based on male mice only, added methodological details for MCAO inclusion criteria, hormone assays, 16S rRNA sequencing, immunofluorescence quantification, and statistical analyses, and expanded the Limitations section to acknowledge the preventive design, lack of infarct-volume measurement, absence of direct HPA-axis manipulation, and limited metabolic phenotyping. All changes have been incorporated into the revised manuscript. Below, we provide a point-by-point response.

Reviewer #1

Comment 1: Line 16-17: "gut-microbiota-HPA-axis axis" is redundant; revise to "gut-microbiota-HPA axis."

Response: We revised this phrase to “gut microbiota–HPA axis.”

Revision:

The phrase has been corrected in the Abstract Line 17.

Comment 2: Lines 20-27: Importance section nicely frames translational relevance, but it essentially repeats the abstract. You could shorten by one or two sentences to avoid redundancy and emphasize what is novel (HFD context, CRH suppression, Akkermansia as a candidate).

Response:
Thank you for this helpful suggestion. We agree that the original Importance section partly repeated the Abstract. We have shortened this section and revised it to emphasize the novel aspects of the study, namely the high-fat diet context, the association between HFD-induced gut dysbiosis and suppressed HPA-axis activity, and *Akkermansia muciniphila* as a candidate microbiota-based intervention. Redundant statements regarding general stroke burden and broad translational implications have been removed.

Revision:

The Importance section has been revised as follows（Lines 21-28）:

High-fat diet-associated metabolic stress is known to worsen stroke outcomes, but how diet-induced gut dysbiosis interacts with neuroendocrine regulation after stroke remains insufficiently understood. The novelty of this study lies in linking HFD-associated microbial disruption with suppressed hypothalamic CRH expression, reduced HPA-axis hormone levels, and aggravated post-stroke inflammation in male mice. We further identify Akkermansia muciniphila as a candidate microbiota-based intervention associated with partial restoration of HPA-axis activity and improved functional recovery after ischemic injury.

Comment 3: Lines 36-39: "a definitive causal link between elevated hormone levels and poor prognosis has not been established" should be supported by more explicit reference to null results or conflicting data, not just . Right now, the logical contrast with the previous sentence is a bit weak.

Response:

We have revised this sentence to more explicitly refer to the inconsistent clinical evidence from corticosteroid treatment studies, thereby clarifying the contrast between observational associations and causal interpretation.

Revision:

Lines 39-41

However, given the inconsistent clinical efficacy of corticosteroid treatment in acute ischemic stroke, a definitive causal link between elevated hormone levels and poor prognosis has not been established.

Comment 4: Lines 52-59: You state that chronic HFD "has been shown to alter the activity of the HPA axis, disrupt glucocorticoid signaling, and potentiate neuroinflammatory cascades" but then say "some inconsistencies remain regarding the direction and extent of these hormonal changes." It would help to very briefly (1-2 clauses) clarify which studies show increased vs decreased HPA activity to pre-emptively address the apparent inconsistency.

Response:

We have revised this sentence to briefly clarify the apparent inconsistency.

Revision:

Lines 59-62

Although some studies have reported increased basal or stress-induced glucocorticoid output after HFD exposure, others have observed blunted HPA-axis responsiveness or reduced

corticosterone levels under prolonged metabolic stress; therefore, inconsistencies remain regarding the direction and extent of these hormonal changes.

Comment 5: Lines 60-62: Use of "--" is nonstandard; replace with a single long dash ("-") or standard punctuation. Look like AI writing.

Response:

Response: Thank you for pointing this out. We have replaced the nonstandard double dashes with a single long dash and checked the manuscript for similar formatting issues.

Revision:

Line 63

Moreover, obesity and metabolic syndrome-key consequences of chronic HFD-have been increasingly recognized

Comment 6: Lines 69-72: The knowledge gap is well-stated ("direct experimental evidence delineating how HFD-induced gut microbiota alterations impact the HPA axis and stroke outcomes remains limited"), but the last sentence of the paragraph slightly overpromises; make clear that you will explore association and partial mechanistic inference rather than directly proving causality.

Response:

Thank you for this helpful suggestion. We agree that the original sentence could be interpreted as implying direct causality. We have revised the final sentence of this paragraph to clarify that the present study examines associations between microbiota-targeted interventions, HPA-axis activity, endogenous hormone levels, and post-stroke outcomes, and provides only partial mechanistic insight rather than direct causal proof.

Revision:

Lines 74-77

To address these gaps, **the present study was designed to examine whether microbiota-targeted interventions in HFD-fed mice are associated with changes in HPA-axis activity, endogenous hormone levels, and post-stroke outcomes, thereby providing partial mechanistic insight into the gut microbiota–HPA-axis–stroke relationship.**

Comment 7: Lines 75-82: The Akkermansia rationale is strong but somewhat scattered. Consider tightening to one compact argument: metabolic benefits, barrier integrity, and prior evidence for HPA modulation, then lead directly into your hypothesis.

Response:

Response: Thank you for this helpful suggestion. We have tightened the rationale for *Akkermansia muciniphila* into a more compact argument. The revised paragraph now first

summarizes its metabolic and gut-barrier benefits, then notes prior evidence for HPA-axis modulation, and finally leads directly to our hypothesis that *Akkermansia* may act as a candidate microbiota-based intervention under HFD conditions.

Revision:

Lines 77-82

Among gut microbes, ***Akkermansia muciniphila*, a mucin-degrading bacterium enriched in healthy individuals, has emerged as an important regulator of metabolic homeostasis and intestinal barrier integrity. Previous studies have shown that *Akkermansia* supplementation can ameliorate obesity-related metabolic dysfunction, reduce inflammation, and modulate gut–brain signaling, including HPA-axis responses under stress-related conditions. Therefore, we hypothesized that *Akkermansia* may serve as a candidate microbiota-based intervention associated with improved neuroendocrine balance and reduced stroke-related injury under high-fat dietary conditions.**

Comment 8: Lines 84-86: "precise, controllable, and low-risk intervention strategy" feels overstated for a preclinical mouse study. Consider softening to "a potentially precise and controllable adjunctive strategy warranting further preclinical and clinical evaluation."

Response:

We agree that the original wording overstated the translational implications of a preclinical mouse study. We have softened this statement and now describe microbiota modulation as a potentially precise and controllable adjunctive strategy that requires further preclinical and clinical evaluation.

Revision:

Lines 86-88

This work aims to deepen our mechanistic understanding of gut microbiota-neuroendocrine interactions and to offer a **potentially precise and controllable adjunctive strategy warranting further preclinical and clinical evaluation.**

Comment 9: Lines 89-101: The two-part design is clear, but you never explicitly state whether there is an HFD group without stroke in the first part for baseline HPA measurements or microbiota assessment. Clarify which groups exist in each part and whether there are non-MCAO controls.

Response:

Thank you for pointing this out. We have clarified in the revised Methods that no separate sham-operated group was included, because the primary aim of the study was to examine the effects of HFD and microbiota intervention under ischemic conditions. We also clarified that

baseline hormone and microbiota measurements were obtained before MCAO.

Revision:

Lines 94-96.

In the first part, ······and HPA axis activity. **Because the primary aim was to examine the effects of HFD and microbiota intervention under ischemic conditions, no separate sham-operated group was included.** Blood samples for hormone assays were collected before MCAO and at 0.5, 1, 2, 4, and 6 hours after occlusion to evaluate dynamic hormonal responses to ischemic stress.

Comment 10: Lines 101-110: Group sizes are given (n=10 behavioral, n=10 histology per group), but it is not clear if this is per cohort or total across experiments. Clarify whether experiments were performed once or repeated and pooled.

Response:

We agree. We have clarified that the reported sample sizes represent the final total number of animals included in each analysis, rather than numbers per repeated cohort. We also specified that behavioral and histology/biochemistry analyses were performed in separate subgroups of mice, with n=10 per group for each subgroup.

Revision:

Lines 104-107.

For each experimental group (ND, HFD, HFDPBS, HFDFMT, and HFDAKK), **behavioral and histology/biochemistry analyses were performed in separate subgroups of mice, with n=10 per group for each subgroup. The reported sample sizes represent the final total number of animals included in each analysis, rather than numbers per repeated cohort.**

Comment 11: Lines 109-110: You state "12 mice per group were randomly selected for 16S rRNA sequencing." Clarify whether these 12 are from the same pool as the n=20 per group, and if so, how overlap with behavioral or histology subgroups was handled (e.g., did some animals contribute both behavior and microbiome data?).

Response: Thank you for this important point. We have clarified how the 16S rRNA sequencing samples overlapped with the behavioral and histological/biochemical subgroups. Fecal samples were collected from all animals before MCAO, and 12 mice per group were randomly selected from the same experimental pool for microbiome analysis. Therefore, some animals contributed both microbiome data and behavioral or histological/biochemical data. Because fecal collection was performed before MCAO and did not interfere with later behavioral testing or tissue collection, this overlap was not expected to influence the outcome measurements. We have also clarified that the microbiome data were analyzed at the group

level and were not treated as an independent validation cohort or used to make animal-level causal inferences.

Revision:

Lines 114-116.

Fecal samples were collected one day before MCAO, and 12 mice per group were randomly selected for 16S rRNA sequencing to analyze gut microbiota composition. **Some animals therefore contributed both microbiome data and behavioral or histological/biochemical data, but fecal collection was performed before MCAO and did not interfere with subsequent outcome assessments.**

Comment 12: Lines 111-120: Sex is limited to male mice, which you acknowledge later in the Limitations. It may be worth pre-emptively mentioning here that only males were used and that rationale will be discussed.

Response:

Thank you for this helpful suggestion. We have added a sentence in the Animals and ethics statement section to explicitly state that only male mice were used, in order to reduce potential variability associated with estrous cycle-related hormonal fluctuations. We also indicate that the implications of this sex limitation are discussed in the Limitations section.

Revision:

Lines 119-121

Six-week-old C57BL/6J mice (18-22 g at baseline; Vital River Laboratory Animal Technology, Beijing, China) were used. **Only male mice were used in this study to reduce potential variability associated with estrous cycle-related hormonal fluctuations; the implications of this sex limitation are discussed in the Limitations section.**

Comment 13: Lines 119-120: You state that allocation and assessment were blinded, but you do not describe the randomization procedure (e.g., block randomization, random number generator) or whether cage effects were controlled. Adding a brief description would strengthen rigor.

Response:

We have added a brief description of the randomization procedure. We also retained the statement that outcome assessments and statistical analyses were performed blinded to group allocation.

Revision:

Lines 128-130

Animals were randomly allocated to experimental groups using a random number generator.

Investigators conducting behavioral scoring, histological assessments, and statistical analyses were blinded to group allocation.

Comment 14: Lines 122-131: Criteria for successful MCAO is "reduction in cerebral blood flow to below 60% of the baseline level, maintained for 60 minutes." You do not state whether animals failing to meet this criterion were excluded and, if so, how many. A CONSORT-style statement on exclusions is important (e.g., number excluded for surgical reasons, death, failure in flow reduction).

Response:
Thank you for this important suggestion. We have added the exclusion numbers for animals excluded because of unsuccessful MCAO model establishment or death during the surgical procedure.

Revision:
Lines 141-142

A reduction in cerebral blood flow to below 60% of the baseline level, maintained for 60 minutes, was considered indicative of successful model establishment. **Across all MCAO experiments, 10 mice were excluded because of unsuccessful model establishment, and 11 mice were excluded because of death during the surgical procedure.**

Comment 15: Lines 132-137: You measure CRH in plasma, which is unconventional and technically challenging; it is important to specify (1) whether this is serum or plasma, (2) validation of the CRH assay in mouse samples (e.g., detection limit, cross-reactivity), and (3) whether samples were run in duplicate/triplicate and intra/inter-assay CV. As CRH is a central mechanistic endpoint, analytical reliability is critical.

Response and revision:
Thank you for this important comment. We agree that the analytical reliability of CRH measurement is critical. We have clarified that hormone levels were measured in serum rather than plasma, and that all ELISA measurements were performed in duplicate. In addition, because circulating CRH levels can be low and variable in serum, we also assessed hypothalamic CRH expression by immunofluorescence staining as a central readout to support the peripheral hormone measurements.

Comment 16: Lines 155-161: You describe collection and sequencing platform, but important details are missing:

Which 16S region (e.g., V3-V4) and primer set were used?

How were reads processed (e.g., DADA2, QIIME2)?

Which reference database and taxonomy classifier?

Were rarefaction depths standardized across samples?

Without these information, reproducibility is limited.

Response:
Thank you for this helpful suggestion. We have expanded the Gut Microbiome Analysis section to include the amplified 16S region and primer set, read-processing pipeline, reference database, taxonomy assignment method, and sequencing-depth normalization. These additions improve the reproducibility of the microbiome analysis.

Revision:
Lines 170-180

Gut microbiota profiling was conducted through 16S rRNA amplicon sequencing on the Illumina NovaSeq platform by Hebei LabMed Technology Co., Ltd. (Hebei, China). The V4 region of the bacterial 16S rRNA gene was amplified using barcoded 515F/806R primers. Paired-end reads were demultiplexed according to barcode and primer sequences, merged using FLASH v1.2.11, processed with Cutadapt to remove residual primer sequences, and quality-filtered using fastp v0.23.1. Chimeric sequences were removed by comparison with the reference database, and effective tags were denoised using the DADA2 module in QIIME2 to generate amplicon sequence variants. Taxonomic annotation was performed in QIIME2 against the SILVA 138.1 database. All samples were normalized to the minimum sequencing depth before alpha- and beta-diversity analyses.

Comment 17: Lines 163-177, 179-188: Methods are fairly detailed, but you do not describe quantification procedures (e.g., how many sections per animal, how many fields per section, selection criteria, and whether thresholding/analysis were done blinded). These details should be stated here, not only in figure legends.

Response:
Thank you for this helpful suggestion. We have added the quantification procedures for immunofluorescence and TUNEL staining, including the number of fields analyzed, ImageJ-based quantification, and blinded analysis.

Revision:

Lines 201-204
For immunofluorescence quantification, three non-overlapping fields per animal were analyzed in predefined regions of interest. Fluorescence intensity was quantified using ImageJ by an investigator blinded to group allocation.

Lines 215-217

For TUNEL quantification, three non-overlapping fields per animal were analyzed in the infarct core. TUNEL-positive cells were expressed as a percentage of DAPI-positive nuclei, and analysis was performed blinded to group allocation.

Comment 18: Lines 190-199: FMT protocol is standard; however you do not mention whether recipient mice received antibiotics prior to FMT, which is common to facilitate colonization. This omission is important because colonization efficiency and interpretation of microbiota changes differ with/without antibiotic preconditioning.

Response:
Response: Thank you for this important comment. We have clarified in the FMT protocol that recipient mice did not receive antibiotic pretreatment before FMT. The intervention was therefore performed in the context of an intact resident microbiota, which should be considered when interpreting colonization efficiency and microbiota changes.

Revision:

Lines 226

Recipient mice did not receive antibiotic pretreatment before FMT

Comment 19: Lines 201-209: You justify AKK dose with a previous study but explicitly state that "The dose-response relationship was not examined in the present study." This should be acknowledged again as a limitation later (dose not optimized, potential under/overdosing).

Response:
We have added this point to the Limitations section, noting that the AKK dose was based on a previous study but was not optimized in the present work, and that potential under- or overdosing cannot be excluded.

Revision:

Limitation

Second, both FMT and AKK treatment were administered before MCAO as preventive interventions. While this design enabled us to explore how microbial modulation influences pre-stroke endocrine status and subsequent outcomes, it does not fully recapitulate the clinical context, where therapeutic interventions typically occur after stroke onset. Therefore, the preventive nature of our design limits its direct translational applicability. **In addition, the AKK dose was selected based on a previous study, but dose-response effects were not examined; therefore, the dose may not represent the optimal therapeutic dose, and potential under- or overdosing cannot be excluded.** Future studies should evaluate post-stroke treatment paradigms to determine the therapeutic potential and optimal intervention

window of microbiota-based strategies.

Comment 20: Lines 227-234: You describe body weight and behavioral deficits; however, absolute body weight values, infarct size, and baseline performance are not reported in text. Consider including key numbers (e.g., mean weight {plus minus} SEM, Longa scores) to give the reader a sense of effect size.

Response:

Thank you for this helpful suggestion. We have added key numerical values to the Results section, including absolute body weight and Longa scores at each post-stroke time point, to provide a clearer sense of effect size.

Revision:

3.1 High-Fat Diet Worsens Post-Ischemic Neurological Outcomes and Is Associated with HPA Axis Dysfunction

To determine whether dietary regimens influenced neurological outcomes after MCAO, we first assessed functional deficits in mice fed distinct diets. As shown in Figure 1a, HFD-fed mice exhibited a significant increase in body weight compared to ND controls **(ND: 28.01 ± 0.47 g; HFD: 33.67 ± 0.85 g)** after 8 weeks of dietary intervention. Neurological function was systematically assessed at days 1, 3, and 7 post-MCAO. The rotarod test revealed significantly impaired motor coordination and endurance in HFD mice compared to ND mice (Figure 1b). **Furthermore, Longa scores were higher in HFD mice than in ND mice at each post-stroke time point (Day 1: ND, 2.20 ± 0.29; HFD, 2.70 ± 0.33; Day 3: ND, 1.70 ± 0.15; HFD, 2.30 ± 0.21; Day 7: ND, 1.00 ± 0.15; HFD, 1.80 ± 0.25), indicating more severe neurological impairment in the HFD group.** (Figure 1c). The open field test showed reduced locomotor activities in HFD mice, evidenced by shorter total movement distances (Figure 1d, e). These results indicate that HFD exacerbates post-ischemic neurological impairment compared to ND.

Comment 21: Lines 237-239: "HFD mice exhibited significantly lower levels of corticosterone, ACTH, and CRH levels at all time points compared to normal diet controls" is strong; indicate whether baseline pre-MCAO hormone levels already differed (they did by design, but emphasize this) and whether there is any time × diet interaction. A simple "at all time points" hides potential differences in dynamic response vs baseline shift.

Response:

Thank you for this helpful comment. The 0 h time point in the hormone curves represents the pre-MCAO baseline level. Thus, HFD mice already showed lower corticosterone, ACTH, and CRH levels before ischemic injury, and this baseline difference was maintained across the

post-MCAO time points. This baseline alteration was the main reason why, in the subsequent FMT and AKK intervention experiments, we focused primarily on pre-MCAO baseline hormone levels to assess whether microbiota intervention could restore the HPA-axis endocrine state before stroke. We agree that the phrase “at all time points” should be interpreted as reflecting both a baseline shift and post-MCAO group differences, rather than a separate conclusion about time × diet interaction.

Comment 22: Lines 241-243: Correlation R^2 values (0.35 and 0.22) show modest relationships; mention that correlations, while significant, explain only a portion of variance. Avoid overinterpreting as strong predictive relationships.

Response:

Thank you for this helpful comment. We have revised the Results section to clarify that, although the correlations between HPA-axis hormone levels and Longa scores were statistically significant, the R^2 values indicate only modest explanatory power. We have therefore avoided interpreting these associations as strong predictive relationships.

Revision:

Lines 276-278

Although these correlations were statistically significant, the R^2 values indicate that CRH and corticosterone levels explained only a modest proportion of the variance in neurological deficit scores.

Comment 23: Lines 261-264: You conclude that reduced hypothalamic CRH expression "suggests" impaired HPA output. This is plausible but you do not distinguish between CRH neuron number loss vs reduced expression per neuron. Clarify in the text what exactly was quantified (cell counts vs mean fluorescence intensity) and whether region boundaries were precisely defined.

Response:

Thank you for this helpful comment. We have clarified that hypothalamic CRH was quantified as mean fluorescence intensity within a predefined hypothalamic region of interest, rather than by counting CRH-positive neurons. We have also softened the interpretation to state that reduced CRH immunoreactivity suggests decreased regional CRH expression, while acknowledging that this analysis cannot distinguish reduced expression per neuron from changes in CRH-positive cell number.

Revision:

Lines 284-286

Immunofluorescence staining for CRH in the hypothalamus revealed markedly reduced CRH expression in HFD-fed mice compared to those fed a normal diet. This observation suggests

that HFD may impair the functional output of the HPA axis by downregulating hypothalamic CRH expression, potentially affecting stress responsiveness and hormonal regulation, **but this analysis does not distinguish reduced expression per neuron from changes in CRH-positive cell number.**

Comment 24: Lines 269-275: Microglial and TUNEL data are described qualitatively but again without numerical values or effect sizes. Adding the magnitude of changes (% increase in Iba-1 intensity, fold change in TUNEL+ cells) would help readers assess biological relevance.

Response:

Thank you for this helpful suggestion. We have added numerical values and fold-change estimates for Iba-1 fluorescence intensity and TUNEL-positive cells to better indicate the magnitude and biological relevance of these effects.

Revision:

Lines 291-292

The HFD group exhibited significantly higher Iba-1 fluorescence intensity compared to the ND group **(1.45 ± 0.12 vs. 1.00 ± 0.06)**

Lines 296-297

The number of TUNEL-positive cells was markedly elevated in HFD-fed mice relative to ND controls **(0.67 ± 0.07 vs. 0.47 ± 0.03)**

Comment 25: Lines 291-297: You show that HFD reduces alpha diversity and alters beta diversity. It would be useful to explicitly state which diversity indices show significant differences (Chao1 and observed features are mentioned, but p-values are not given in text).

Response:

Thank you for this helpful suggestion. We have supplemented the corresponding p-values for the alpha-diversity indices in the Results section. We have also updated Figure 3 to include additional alpha-diversity measures, including Chao1, observed features, Shannon, and Simpson indices, to more clearly show the effects of HFD on gut microbial diversity.

Revision:

We revised Figure 3 and added the requested alpha-diversity plots, including Chao1, observed features, Shannon, and Simpson indices.

To investigate the impact of a high-fat diet on the gut microbiota of mice, fecal samples were collected one day prior to MCAO surgery for 16S rRNA amplicon sequencing analysis. The

Venn diagram revealed a total of 382 ASVs shared between the HFD and ND groups, with 1,174 unique ASVs identified in the ND group and 840 unique ASVs in the HFD group (Figure 3b). Alpha diversity analysis indicated that microbial richness was notably reduced in HFD-fed mice compared to those on a normal diet, **as reflected by lower Chao1 (p=0.0004), observed features (p=0.004), Shannon index (p=0.016), and Simpson index (p= 0.008) (Figure 3a).** Beta diversity analysis further demonstrated significant differences in microbial community composition between the two groups (Figure 3d). These results suggest that 8 weeks of HFD feeding markedly reduced both the richness and diversity of the gut microbiota in mice. Multilevel taxonomic analysis of microbial composition revealed that, at the phylum level, the relative abundances of Firmicutes, Actinobacteria, and Desulfobacterota were significantly increased in the HFD group, whereas the abundances of Verrucomicrobiota and Lactobacillota were significantly decreased compared to the ND group (Figure 3d,e). Further analysis at the genus level showed a significant reduction in the abundance of Akkermansia in the HFD group relative to the ND group (Figure 3d,f). Collectively, these findings indicate that the gut microbial composition in HFD-fed mice differs markedly from that in mice maintained on a normal diet.

Comment 26: Lines 299-301: The phylum-level shifts (increased Firmicutes, Actinobacteria, Desulfobacterota; decreased Verrucomicrobiota, Lactobacillota) are described but without mentioning statistical test or correction; clarify that these differences are statistically significant and indicate whether you controlled for multiple testing

Response:

Response: Thank you for this helpful suggestion. We have revised Figure 3 to include quantitative comparisons of representative bacterial phyla and genera, rather than only showing stacked bar plots.

Revision:

Figure3

We revised Figure 3 by adding quantitative analysis plots for representative bacterial phyla and genera.

Comment 27: Lines 302-304: Akkermansia reduction is central to the intervention concept; consider quantifying its relative abundance (e.g., median %, IQR) to emphasize the magnitude of depletion.

Response:

We have revised Figure 3 to include quantitative comparisons of representative bacterial phyla and genera, including Akkermansia, rather than only showing stacked bar plots.

Revision:

Figure3

We revised Figure 3 by adding quantitative analysis plots for representative bacterial phyla and genera, including *Akkermansia*.

Comment 28: Lines 325-325: HFD mice receiving FMT or AKK had reduced body weight compared with HFDPBS. This is important because it introduces a potential confounder: improved outcomes may partially reflect weight loss/metabolic improvement rather than solely microbiota-HPA effects. You should acknowledge this in the Results (not only Discussion) and ideally adjust or at least discuss in terms of metabolic parameters.

Response:

Thank you for this important comment. We agree that the reduced body weight after FMT or AKK administration may represent a potential confounder. We have added a statement in the Results section to acknowledge that the improved hormone levels and stroke outcomes should be interpreted in the context of concomitant metabolic changes. We have also expanded the Limitation section to note that future studies with weight-matched controls and additional metabolic parameters are needed to disentangle microbiota-HPA-axis effects from general metabolic improvement.

Revision:

Lines 335-337

Because both FMT and AKK administration reduced body weight in HFD mice, the subsequent improvements in hormone levels and stroke outcomes should be interpreted in the context of concomitant metabolic changes, rather than being attributed solely to microbiota-HPA-axis modulation.

Limitation

Besides, the reduction in body weight after FMT or AKK administration represents a potential confounding factor, as improved neurological outcomes may partly reflect weight loss or broader metabolic improvement.

Comment 29: Lines 327-333: You state that ACTH and corticosterone are significantly elevated, whereas CRH increases but does not reach significance in HFDFMT. This suggests that CRH might not be the primary driver of peripheral hormone changes, or that power is limited; you should note this nuance explicitly and avoid presenting CRH restoration as equally robust in all groups later.

Response:

We agree that the CRH result in the HFD^{FMT} group should be interpreted more cautiously. We have revised the Results section to explicitly state that FMT significantly increased ACTH and corticosterone levels, whereas serum CRH showed only a non-significant increasing trend. We have also revised later descriptions to avoid presenting CRH restoration as equally robust across all intervention groups, and now describe the FMT effect as a partial restoration of HPA-axis hormone profiles.

Revision:

Lines 345-346
Thus, FMT appeared to partially restore the HPA-axis hormone profile, with more robust effects on ACTH and corticosterone than on serum CRH.

Comment 30: Lines 335-342: Longa and rotarod improvements are described, with AKK showing earlier, stronger effects than FMT. It would help to state effect sizes (e.g., mean difference in Longa score vs HFDPBS on day 7) and mention whether there were interactions between time and treatment (since improvements emerge over days).

Response:
Thank you for this helpful suggestion. We have revised the Results section to include representative effect-size information for the behavioral outcomes, focusing on the Day 7 differences in Longa score and rotarod latency relative to HFDPBS controls.

Revision:

Behavioral performance was evaluated at 1, 3, and 7 days after reperfusion. Longa neurological scores showed that both HFDFMT and HFDAKK groups had significantly lower scores than HFDPBS controls, reflecting improved neurological recovery (Figure 4f). **This difference was evident on day 7, when Longa scores were reduced by 0.70 points in the HFDFMT group and 0.90 points in the HFDAKK group compared with HFDPBS mice.** In the rotarod test, latency to fall was significantly prolonged in the HFDAKK group at all examined time points, indicating consistently enhanced motor coordination. In contrast, the HFDFMT group exhibited a trend toward improvement during the early phase, which became statistically significant on day 7. **At this time point, rotarod latency was increased by 63.3 s in HFDFMT mice and 84.5 s in HFDAKK mice compared with HFDPBS controls (Figure 4g)**. No significant differences were found between the HFDAKK and HFDFMT groups in either neurological scores or rotarod performance at any time point.

Open-field testing further supported these behavioral results. Representative trajectory plots and heatmaps revealed greater spontaneous locomotor activity in both intervention groups compared with HFDPBS controls. Quantitative analysis showed that total movement distance was significantly greater in the HFDAKK group at all time points after MCAO, whereas the

HFDFMT group showed increased locomotion with a statistically significant difference on day 7 (Figure 4h–i). **On day 7, total movement distance was approximately doubled in the HFDFMT group and increased by more than two-fold in the HFDAKK group relative to HFDPBS controls.**

Comment 31: Lines 349-352: You state "Although elevated HPA-axis hormones accompanied functional improvement, the present data do not establish a causal relationship." This is an important caveat and might be moved forward in the paragraph to prevent readers from inferring causality earlier.

Response:
Thank you for this helpful suggestion. We agree that this caveat should be stated earlier to avoid overinterpretation. We have moved this statement forward in the paragraph and revised the wording to clarify from the outset that the observed increase in HPA-axis hormones accompanied, but does not prove causally mediate, the improved behavioral outcomes.

Revision:

Lines 349-353

Although elevated HPA-axis hormones accompanied functional improvement, the present data do not establish a causal relationship between hormone restoration and behavioral recovery. Therefore, the improved outcomes after FMT or AKK administration should be interpreted as being associated with, rather than directly caused by, changes in HPA-axis activity.

Lines 373-375

Together, these findings suggest that FMT and AKK administration were associated with improved post-stroke functional recovery and partial restoration of HPA-axis hormone profiles in HFD mice, with AKK showing relatively earlier and more consistent behavioral effects.

Comment 32: Lines 371-376: You refer to "Figure S1a-c" for CRH restoration after FMT and AKK, but these are supplementary figures; given that CRH is a central mechanistic link, consider moving these data into main figures or at least referencing them more strongly.

Response:
Thank you for this helpful suggestion. We agree that hypothalamic CRH expression is an important mechanistic readout in this study. We have revised the main text to refer more explicitly to these data and to emphasize that FMT and AKK administration increased hypothalamic CRH immunoreactivity, supporting partial restoration of central HPA-axis

activity. We retained these images in the Supplementary Figure to avoid overloading the main figures, but strengthened their description in the Results section.

Revision:

Lines 382-384

First, consistent with the peripheral hormone results, hypothalamic CRH immunofluorescence further showed increased CRH immunoreactivity after FMT and AKK administration, supporting partial restoration of central HPA-axis activity in HFD mice.

Comment 33: Lines 378-383: Iba-1 and TUNEL changes are presented, but the phrase "more notable reduction" and "a decreasing trend (p = 0.0574)" risks overemphasizing near-significant results. It is better to state clearly that TUNEL reduction in HFDFMT did not reach significance and treat it as hypothesis-generating rather than quasi-positive.

Response:

We agree that the previous wording may have overemphasized the near-significant TUNEL result in the HFD^FMT group. We have revised the Results section to clearly state that TUNEL reduction after FMT did not reach statistical significance.

Revision:

Lines 395-398

whereas the reduction in the HFDFMT group did not reach statistical significance (p=0.0574). These findings suggest that AKK administration may mitigate post-ischemic neuronal apoptosis, whereas the potential anti-apoptotic effect of FMT requires further confirmation (Figure S2a–c).

Comment 34: Lines 399-402: Alpha diversity is reduced in intervention groups compared with HFDPBS. This is a paradoxical finding given that interventions are beneficial; you should highlight this tension more clearly and avoid implying that diversity restoration is the mechanism of benefit.

Response:

Thank you for this important comment. We agree that the reduced alpha diversity after FMT or AKK administration should not be interpreted as diversity restoration. We have revised the Discussion to explicitly highlight this apparent tension and to clarify that the beneficial effects of the interventions are more likely related to specific compositional or functional microbial changes, rather than an overall increase in alpha diversity.

Revision:

Discussion

The observed associations, although statistically significant, do not confirm direct endocrine causality. **Notably, alpha diversity was reduced after FMT and AKK administration compared with HFDPBS mice, despite the beneficial effects of these interventions on hormone levels and post-stroke outcomes. Therefore, these findings should not be interpreted as evidence that microbiota intervention improved recovery by restoring overall microbial diversity. Instead, the protective effects may be more closely related to specific compositional or functional changes, such as Akkermansia-associated alterations and modulation of gut-brain neuroendocrine signaling.** Prior evidence supports the notion that microbial components and metabolites can influence HPA axis regulation

Comment 35: Lines 404-406: The phylum-level differences in HFDAKK (higher Proteobacteria) might raise concerns about potential pro-inflammatory consequences; this deserves explicit mention and cautious interpretation.

Response:

We have revised the Discussion to explicitly acknowledge the increased relative abundance of Proteobacteria in the HFD^AKK group and to interpret this finding cautiously. We now note that, although AKK administration was associated with improved behavioral and neuroendocrine outcomes, its effects on microbial composition are complex and should not be interpreted as uniformly beneficial.

Revision:

Discussion

In addition, the increased relative abundance of Proteobacteria in the HFDAKK group should be interpreted cautiously, as expansion of this phylum is often associated with inflammatory or dysbiotic states. Although AKK administration improved behavioral and neuroendocrine outcomes in the present study, this compositional change indicates that its microbiota-level effects are complex and not uniformly beneficial.

Comment 36: Lines 420-429: The summary of findings is accurate but somewhat overstated in mechanistic certainty ("interventions...partially restored CRH expression..., elevated systemic glucocorticoid levels, and improved...outcomes"). You should reiterate that causality is not demonstrated and that HPA changes may be one of several mediating pathways.

Response:

We agree that the mechanistic interpretation should be more cautious. We have revised the Discussion to clarify that FMT and AKK administration were associated with, rather than proven to cause, partial restoration of HPA-axis activity and improved outcomes.

Revision:

Lines 424-428

More importantly, **our findings show that FMT and AKK administration were associated with partial restoration of hypothalamic CRH immunoreactivity, increased circulating HPA-axis hormone levels, and improved post-stroke functional outcomes in HFD mice. However, these data do not establish a causal relationship between HPA-axis changes and functional recovery.**

Comment 37: Lines 435-446: You nicely acknowledge that association does not prove endocrine causality. However, later sentences (e.g., lines 441-443) risk reintroducing a causal tone ("may serve...as modulators of central neuroendocrine circuits") without clearly framing it as speculative. Consider adding explicit wording like "We speculate that..." or "It is plausible but remains unproven that...".

Response:

We have revised the wording to explicitly frame the proposed modulation of central neuroendocrine circuits as speculative, using "We speculate that" to avoid implying direct mechanistic proof.

Revision:

Lines 448

We speculate that specific microbial taxa, such as Akkermansia, may serve not only as anti-inflammatory agents but also as modulators of central neuroendocrine circuits.

Comment 38: Lines 448-459: The discussion of endocrine-independent pathways is strong, but you may want to explicitly highlight that, given modest corticosterone changes and strong anti-inflammatory effects, non-HPA mechanisms may account for a substantial portion of benefit.

Response:

We have revised the Discussion to more explicitly acknowledge that non-HPA mechanisms may contribute substantially to the beneficial effects of FMT and AKK administration, particularly given the modest changes in corticosterone and the stronger anti-inflammatory and anti-apoptotic effects observed in the study.

Revision:

Lines 461-463

Given the modest changes in circulating corticosterone and the pronounced reduction in inflammatory and apoptotic markers, non-HPA mechanisms may account for a substantial portion of the observed benefit after FMT or AKK administration.

Comment 39: Lines 462-469: You discuss reduced alpha diversity in interventions but do not critically reflect on whether this may carry risks or reflect dominance of a few taxa (including Proteobacteria). Adding a short caveat would demonstrate balanced interpretation.

Response:

We have added a short caveat to the Discussion noting that reduced alpha diversity after intervention may reflect dominance of selected taxa, including Proteobacteria, and that the potential consequences of this compositional shift should be interpreted cautiously.

Revision:

Lines 474-475

While alpha diversity was reduced in the intervention groups-perhaps due to the selective colonization of dominant taxa-β diversity analyses indicated a clear shift in overall composition. **This reduced diversity may reflect dominance of selected taxa, including Proteobacteria, and its potential long-term consequences require cautious interpretation.**

Comment 40: Lines 471-472: Suggesting gnotobiotic and defined consortia approaches is appropriate; you might also suggest metabolomics and targeted SCFA or bile acid analysis as next steps.

Response:

We have revised the Discussion to include metabolomics and targeted SCFA or bile acid analyses as important future directions, in addition to gnotobiotic models and defined microbial consortia.

Revision:

Lines 482-484

Future studies using gnotobiotic models, defined microbial consortia, metabolomics, and targeted SCFA or bile acid analyses will be needed to identify the specific microbial metabolites and pathways involved.

Comment 41: Lines 474-482: The idea that patients with suppressed HPA axis may benefit from microbiota-based approaches is interesting but highly speculative at this stage. You should explicitly say that clinical HPA suppression in obese/MetS stroke patients has not yet been systematically linked to microbiota signatures in the way your model suggests, and that translation will require substantial validation.

Response:

We have revised the Discussion to clarify that this clinical implication remains speculative

and that substantial clinical validation will be needed before microbiota-based approaches can be translated to obese or metabolic syndrome stroke patients.

Revision:

Lines 495-498

However, this clinical implication remains speculative. HPA-axis suppression in obese or metabolic syndrome stroke patients has not yet been systematically linked to specific gut microbiota signatures, and substantial clinical validation will be needed before translation.

Comment 42: Lines 478-481: The sentence beginning "Moreover, Emerging concepts in stroke medicine..." has capitalization and stylistic issues ("Emerging" should not be capitalized; "Moreover," is unnecessary).

Response:

Thank you for pointing this out. We have corrected the capitalization issue and removed the unnecessary “Moreover,” to improve the style and readability of the sentence.

Revision:

Lines 498

remove “Moreover”

Comment 43: Lines 486-495: The sex limitation is well discussed, but you might also highlight that Akkermansia-HPA interactions could be sex-specific, making male-only data particularly hard to generalize.

Response:

We have revised the Limitations section to further emphasize that Akkermansia–HPA-axis interactions may be sex-specific, which limits the generalizability of the present male-only findings.

Revision:

Lines 509-511

Moreover, Akkermansia-HPA axis interactions may be sex-specific, which further limits the generalizability of the present male-only findings.

Comment 44: Lines 497-502: You acknowledge the preventive design; this is appropriate. You might also mention that the 7-day pre-treatment window is relatively short and does not model long-standing microbiome modulation.

Response:

We have revised the Limitations section to note that the 7-day pretreatment window was relatively short and may not fully represent long-standing microbiome modulation.**Revision:**

Lines 519-522

Therefore, the preventive nature of our design, together with the relatively short 7-day pretreatment window, limits its direct translational applicability and may not fully model long-standing microbiome modulation.

Comment 45: Lines 504-507: You mention that mechanistic relationships remain incompletely understood; this could be strengthened by explicitly acknowledging the absence of HPA blockade experiments (e.g., glucocorticoid receptor antagonists, adrenalectomy) and lack of direct CRH neuron manipulation.

Response:
We have revised the Limitations section to clarify that the mechanistic relationship between gut microbial alterations, CRH regulation, and corticosterone secretion remains incompletely understood and requires further confirmation using direct approaches.

Revision:

Lines 533-535
In addition, the mechanistic relationship between gut microbial alterations, CRH regulation, and corticosterone secretion remains incompletely understood a**nd requires further confirmation using direct approaches, such as HPA-axis blockade or targeted modulation of CRH signaling.**

Comment 46: Missing limitation:

No metabolic phenotyping beyond body weight (e.g., glucose, insulin, lipids) is provided, limiting interpretation of how "metabolic stress" was modulated by interventions. Adding a note about missing metabolic endpoints would strengthen transparency.

Antibiotics not used before FMT (if true) could be explicitly acknowledged as a factor that may limit colonization efficiency and generalizability to standard FMT protocols.

Response:
Thank you for these helpful suggestions. We have revised the Limitations section to acknowledge that metabolic phenotyping was limited to body weight and did not include glucose, insulin, lipid profiles, or other metabolic endpoints. We have also explicitly noted that recipient mice did not receive antibiotic pretreatment before FMT, which may have limited donor microbiota engraftment and may affect generalizability to standard FMT protocols.

Revision:

Limitation

In addition, the mechanistic relationship between gut microbial alterations, CRH regulation, and corticosterone secretion remains incompletely understood and requires further confirmation using direct approaches, such as HPA-axis blockade or targeted modulation of CRH signaling. Metabolic phenotyping was also limited to body weight, without glucose, insulin, lipid, or other metabolic endpoints, limiting interpretation of metabolic changes after intervention. Moreover, recipient mice did not receive antibiotic pretreatment before FMT, which may have affected donor microbiota engraftment. Further research integrating metabolic assessment and causal manipulation approaches is required to clarify whether microbiota-induced hormonal changes directly mediate neuroprotection after stroke.

Reviewer 2

Public repository details (Required)： Gut microbiome data need to be deposited.

Response:

Thank you for this requirement. We have added a Data Availability statement and will deposit the 16S rRNA sequencing data in a public repository before publication. The accession number will be provided once the submission to the repository is completed.

Comment 1: The current conclusions, particularly those regarding therapeutic implications for stroke, are overconcluded given that the study relies exclusively on male data. Biological sex is a critical variable in stroke pathophysiology and treatment response; therefore, the results cannot be assumed to apply to female cohorts without direct investigation. The study design was only based on male mice therefore therapeutic implications for stroke patients cannot legitimately apply to female patients on the basis of male-only data. The abstract , discussion and throughout the draft need a revision to explicitly limit all conclusions to male animals.

Response:

Thank you for this important comment. We fully agree that the use of male mice only limits the generalizability of the present findings. We have revised the Abstract, Discussion, Conclusion, and relevant sections throughout the manuscript to explicitly state that our conclusions are based on male mice and should not be assumed to apply to females without direct investigation. We have also expanded the Discussion to address this limitation in more detail, emphasizing that biological sex is a critical variable in stroke pathophysiology, HPA-axis regulation, microbiota composition, and treatment response.

Comment 2: The findings in this study were mainly correlational not a direct causation. I suggest the authors to revise the title, abstract, and discussion to use more conservative, association-based language (e.g., "is associated with" or "correlates with") rather than strongly address a direct cause, which could lead to scientifically misleading.

Response:

Thank you for this important comment. We agree that the present findings are mainly correlational and do not establish direct causality. This issue was also raised by Reviewer 1, and we have revised the title, Abstract, Discussion, Conclusion, and relevant sections throughout the manuscript to use more conservative, association-based language.

Revision:

Lines 349-352

Although elevated HPA-axis hormones accompanied functional improvement, the present data do not establish a causal relationship between hormone restoration and behavioral recovery. Therefore, the improved outcomes after FMT or AKK administration should be interpreted as being associated with, rather than directly caused by, changes in HPA-axis activity.

Lines 373-376

Together, these findings suggest that FMT and AKK administration were associated with improved post-stroke functional recovery and partial restoration of HPA-axis hormone profiles in HFD mice, with AKK showing relatively earlier and more consistent behavioral effects.

Lines 424-428

More importantly, **our findings show that FMT and AKK administration were associated with partial restoration of hypothalamic CRH immunoreactivity, increased circulating HPA-axis hormone levels, and improved post-stroke functional outcomes in HFD mice. However, these data do not establish a causal relationship between HPA-axis changes and functional recovery.**

Lines 448

We speculate that specific microbial taxa, such as Akkermansia, may serve not only as anti-inflammatory agents but also as modulators of central neuroendocrine circuits.

Comment 3: The data shown in the Result section were based only on behavioural scores (Longa, rotarod, open field) and inflammatory markers without establishing whether these differences are accompanied by proportional differences in infarct size. If HFD mice have larger infarcts, some or all of the behavioural deficit could be explained by greater tissue loss rather than impaired recovery. Could the authors add infarct volume as percentage of ipsilateral hemisphere to strengthen this observation?

Response:

Thank you for this helpful suggestion. We agree that infarct volume would provide an

important anatomical measure to further support the behavioral findings. However, infarct volume was not quantified in the present study, and the available tissue sections were primarily used for immunofluorescence and TUNEL analyses. We have therefore added this point to the Limitations section, noting that the absence of infarct volume measurement limits our ability to determine whether behavioral deficits were partly driven by differences in tissue loss.

Revision:

Lines 535-536

Infarct volume was not directly quantified, limiting anatomical interpretation of the behavioral outcomes.

Comment 4: In this study, the authors proposed that gut dysbiosis suppresses CRH through gut-barrier dysfunction and TLR4/NF-κB signalling, or via microbial metabolites, yet none of these intermediate steps were measured. Please elaborate on this matter or revise the draft to conclude only evidence supported by the data.

Response:

Thank you for this comment. We would like to clarify that gut-barrier dysfunction, TLR4/NF-κB signaling, and microbial metabolites were discussed in the Discussion based on prior evidence, rather than presented as mechanisms directly demonstrated in the present study. For example, the paragraph begins with “Prior evidence supports the notion that microbial components and metabolites can influence HPA axis regulation.” To avoid any misunderstanding, we have further revised the wording to make clear that these pathways are literature-based possible mechanisms and were not directly assessed in our model.

Revision:

Lines 441-443

Prior evidence supports the notion that microbial components and metabolites can influence HPA-axis regulation, although these intermediate pathways were not directly assessed in the present study

Comment 5: Iba-1 fluorescence intensity is useful as a proxy for microglial morphological activation. It could not distinguish pro- from anti-inflammatory states (M1/M2 polarisation), or capture infiltrating monocytes or other immune effectors. Therefore, the mentioned exacerbated neuroinflammation was derived based on a single histological marker without systemic inflamatory data. Could the authors further explain or clarify this conclusion?

Response:

Thank you for this important comment. We agree that Iba-1 fluorescence intensity alone

cannot fully characterize neuroinflammation or distinguish microglial polarization states and other immune effectors. We have therefore revised the wording throughout the manuscript to describe these findings more specifically as changes in Iba-1-positive microglial activation, rather than as a comprehensive measure of neuroinflammation.

Comment 6: The main key from this study was that microbiota intervention could restore HPA axis activity and that this hormonal restoration could improved stroke outcomes. This is a bit contradicted by the FMT data. Figure 4e shows that CRH levels in the HFD-FMT group do not significantly differ from HFD-PBS controls ($p > 0.05$; described as a "trend"). Yet the same group shows significantly improved Longa scores, rotarod performance, and open-field distance (Figs. 4f-i) and reduced Iba-1 staining (Fig. 5). If CRH restoration is the mechanism, how did a group without significant CRH elevation achieve equivalent behavioural benefit? Perhaps, the authors can discuss this discrepancy.

Response:
Thank you for this important comment. We agree that the FMT results indicate that behavioral improvement cannot be explained solely by significant serum CRH restoration. We have revised the Discussion to explicitly acknowledge this discrepancy and to clarify that non-HPA mechanisms may also contribute substantially to the observed benefits. Specifically, we now state that, given the modest changes in circulating corticosterone and the pronounced reduction in inflammatory and apoptotic markers, non-HPA mechanisms may account for a substantial portion of the benefit after FMT or AKK administration.

Revision:

Lines 461-463

Given the modest changes in circulating corticosterone and the pronounced reduction in inflammatory and apoptotic markers, non-HPA mechanisms may account for a substantial portion of the observed benefit after FMT or AKK administration.

Comment 7: A single dose of 2×10^9 CFU/200 μL was used throughout, justified by reference to one prior study with a different disease model. Please elaborate more or add rationale why this single dose was selected.

Response:
Thank you for this helpful comment. We have added a rationale and limitation regarding the AKK dose in the Discussion. We now clarify that the dose was selected based on a previous study, but dose-response effects were not examined in the present work. Therefore, this dose may not represent the optimal therapeutic dose, and potential under- or overdosing cannot be excluded.

Revision:

Lines 526-529

The AKK dose was selected based on a previous study, but dose-response effects were not examined; therefore, the dose may not represent the optimal therapeutic dose, and potential under- or overdosing cannot be excluded.

Comment 8: Methods section (lines 163-178) only showed antibody concentrations and excitation/emission wavelengths but omits instrument settings critical for reproducibility of fluorescence intensity comparisons. Please add more details.

Response:
Thank you for this helpful suggestion. We have expanded the Methods section to include additional imaging parameters relevant to fluorescence intensity comparison. Specifically, we clarified that images were acquired using a 40× objective and that exposure time, gain, light intensity, and image resolution were kept constant across groups within the same staining batch.

Revision:

Lines 199-201

Fluorescence images were acquired using a 40× objective. For each marker, exposure time, gain, light intensity, and image resolution were kept constant across all groups within the same staining batch.

Comment 9: Lines 129-131 state the criterion but lines 102-103 present group sizes without accounting for surgical failures. I suggest the authors adding a statement in Methods or Results reporting the number of animals per group that met the CBF success criterion, the number excluded, and the total enrolled. Report whether CBF monitoring was continuous or sampled.

Response:
Response: We have revised the Methods section to clarify animal exclusions during MCAO experiments. Specifically, we now state that, across all MCAO experiments, 10 mice were excluded because of unsuccessful model establishment and 11 mice were excluded because of death during the surgical procedure.

Revision:

Lines 141-142

Across all MCAO experiments, 10 mice were excluded because of unsuccessful model establishment, and 11 mice were excluded because of death during the surgical procedure.

Comment 10: Need bioinformatics analysis details (Line 159-161)

Response:

Response: We have supplemented the bioinformatics analysis details in the corresponding Methods section, including read processing, quality filtering, chimera removal, ASV generation, taxonomic annotation, reference database, and sequencing-depth normalization before diversity analyses.

Revision:

Lines 169-182

Fecal sample collection was performed by elevating the tail and applying gentle abdominal pressure to stimulate defecation, freshly expelled feces were collected using sterile forceps and immediately transferred into sterile cryovials, which were subsequently stored at -80°C. Each biological sample contained 2-3 fecal pellets. Gut microbiota profiling was conducted through 16S rRNA amplicon sequencing on the Illumina NovaSeq platform by Hebei LabMed Technology Co., Ltd. (Hebei, China). The V4 region of the bacterial 16S rRNA gene was amplified using barcoded 515F/806R primers. Paired-end reads were demultiplexed according to barcode and primer sequences, merged using FLASH v1.2.11, processed with Cutadapt to remove residual primer sequences, and quality-filtered using fastp v0.23.1. Chimeric sequences were removed by comparison with the reference database, and effective tags were denoised using the DADA2 module in QIIME2 to generate amplicon sequence variants. Taxonomic annotation was performed in QIIME2 against the SILVA 138.1 database. All samples were normalized to the minimum sequencing depth before alpha- and beta-diversity analyses.

Comment 11: For corticosterone assay, please add a time of day for blood collection of each group.

Response:

Thank you for this helpful suggestion. We have clarified that MCAO procedures were performed between 11:00 and 15:00 and that blood samples were collected at predefined time points using the same schedule across groups.

Revision:

Lines 145-147

MCAO procedures were performed between 11:00 and 15:00, and blood samples were collected at predefined time points using the same schedule across groups.

Conclusion

We sincerely thank the reviewers for their constructive and insightful comments. In the revised manuscript, we have substantially improved the clarity and rigor of the study by

limiting conclusions to male mice, using more conservative association-based language, adding methodological details, reporting additional quantitative results, and expanding the discussion of key limitations. We believe these revisions have strengthened the manuscript and improved the transparency and interpretation of our findings.

Re: Spectrum03549-25R2 (**Gut Dysbiosis Impairs HPA Axis Function and Exacerbates Post-Stroke Inflammation in High-Fat Diet-Fed Male Mice**)

Dear Dr. Yunpeng Liu:

Your manuscript has been accepted, and I am forwarding it to the ASM production staff for publication. Your paper will first be checked to make sure all elements meet the technical requirements. ASM staff will contact you if anything needs to be revised before copyediting and production can begin. Otherwise, you will be notified when your proofs are ready to be viewed.

Data Availability: ASM policy requires that data be available to the public upon online posting of the article, so please verify all links to sequence records, if present, and make sure that each number retrieves the full record of the data. If a new accession number is not linked or a link is broken, provide production staff with the correct URL for the record. If the accession numbers for new data are not publicly accessible before the expected online posting of the article, publication may be delayed; please contact ASM production staff immediately with the expected release date.

Publication Fees: For information on publication fees and which article types have charges, please visit our website. We have partnered with Copyright Clearance Center (CCC) to collect author charges. If fees apply to your paper, you will receive a message from no-reply@copyright.com with further instructions. For questions related to paying charges through RightsLink, please contact CCC at ASM_Support@copyright.com or toll free at +1-877-622-5543. CCC makes every attempt to respond to all emails within 24 hours.

ASM Membership: Corresponding authors may join or renew ASM membership to obtain discounts on publication fees. Need to upgrade your membership level? Please contact Customer Service at Service@asmusa.org.

Embargo Policy: A press release may be issued as soon as the manuscript is posted on the Spectrum Latest Articles webpage. The corresponding author will receive an email with the subject line "ASM Journals Author Services Notification" when the article is available online.

Cover Image Submissions: If you would like to submit a potential Cover Image, please email a file and a short legend to spectrum@asmusa.org. Please note that we can only consider images that (i) the authors created or own and (ii) have not been previously published. By submitting, you agree that the image can be used under the same terms as the published article. Image File requirements: TIF/EPS, 7.5 inches wide by 8.25 inches tall (at least 2,250 pixels wide by 2,475 pixels tall), minimum 300 dpi resolution (600 dpi preferred), RGB, and no figure elements, e.g., arrows or panel labels. The legend should be a short description of the image, 1-2 sentences recommended. Please use the ASM Journals Cover Image Sizing Tool (https://journals.asm.org/pb-assets/html/asm-cover-sizing-tool.html) to ensure that your proposed cover image meets our size requirements.

The ASM Journals program strives for constant improvement in our submission and publication process. Please tell us how we can improve your experience by taking this quick Author Survey.

Thank you for submitting your paper to Spectrum.

Sincerely,
Wanilada Rungrassamee
Editor
Microbiology Spectrum
