# Supplementary material for: Gut dysbiosis impairs HPA axis function and exacerbates post-stroke inflammation in high-fat diet-fed male mice
Source: Microbiol Spectr. 2026 Aug 21;14(10):e03549-25. doi: 10.1128/spectrum.03549-25 (PMC13689323; doi:10.1128/spectrum.03549-25)
Supplement: Supplementary file 2 [file spectrum.03549-25-s0001.docx]

**Figure S1.** Gut microbiota intervention regulates hypothalamic CRH expression. (a) Representative immunofluorescence staining of CRH (red) in each group. (b) Analysis was performed in the hypothalamic region. (c) Normalized quantification of mean fluorescence intensity of CRH across groups, using the HFD^PBS^ group as the control. n=5 per group. *, *P*<0.05, **, *P*<0.01, ***, *P*<0.001.

**Figure S2.** Gut microbiota intervention alleviates neuronal apoptosis after MCAO. (a) Representative immunofluorescence staining of TUNEL (green) in each group. (b) Analysis was performed in the infarct region. (c) Quantification of the percentage of TUNEL-positive cells across groups. n=5 per group. *, *P*<0.05, **, *P*<0.01, ***, *P*<0.001.
